# Heparan Sulfate Organizes Neuronal Synapses through Neurexin Partnerships

**DOI:** 10.1016/j.cell.2018.07.002

**Published:** 2018-09-06

**Authors:** Peng Zhang, Hong Lu, Rui T. Peixoto, Mary K. Pines, Yuan Ge, Shinichiro Oku, Tabrez J. Siddiqui, Yicheng Xie, Wenlan Wu, Stephanie Archer-Hartmann, Keitaro Yoshida, Kenji F. Tanaka, A. Radu Aricescu, Parastoo Azadi, Michael D. Gordon, Bernardo L. Sabatini, Rachel O.L. Wong, Ann Marie Craig

**Affiliations:** 1 Djavad Mowafaghian Centre for Brain Health and Department of Psychiatry, University of British Columbia, Vancouver, BC V6T 2B5, Canada; 2 Howard Hughes Medical Institute, Harvard Medical School, Department of Neurobiology, Boston, MA 02115, USA; 3 Istituto Italiano di Tecnologia, Genova 16163, Italy; 4 Department of Zoology and Life Sciences Institute, University of British Columbia, Vancouver, BC V6T 1Z3, Canada; 5 Medical School, Henan University of Science and Technology, Luoyang 471023, China; 6 Complex Carbohydrate Research Center, University of Georgia, Athens, GA 30602, USA; 7 Department of Neuropsychiatry, Keio University School of Medicine, Tokyo 160-8582, Japan; 8 MRC Laboratory of Molecular Biology, Cambridge Biomedical Campus, Cambridge CB2 0QH, UK; 9 Department of Biological Structure, University of Washington, Seattle, WA 98195, USA

**Keywords:** synaptogenesis, synaptic transmission, synaptic adhesion protein, proteoglycan, heparan sulphate, neurexin, neuroligin, LRRTM, mossy fiber, thorny excrescence

## Abstract

Synapses are fundamental units of communication in the brain. The prototypical synapse-organizing complex neurexin-neuroligin mediates synapse development and function and is central to a shared genetic risk pathway in autism and schizophrenia. Neurexin’s role in synapse development is thought to be mediated purely by its protein domains, but we reveal a requirement for a rare glycan modification. Mice lacking heparan sulfate (HS) on neurexin-1 show reduced survival, as well as structural and functional deficits at central synapses. HS directly binds postsynaptic partners neuroligins and LRRTMs, revealing a dual binding mode involving intrinsic glycan and protein domains for canonical synapse-organizing complexes. Neurexin HS chains also bind novel ligands, potentially expanding the neurexin interactome to hundreds of HS-binding proteins. Because HS structure is heterogeneous, our findings indicate an additional dimension to neurexin diversity, provide a molecular basis for fine-tuning synaptic function, and open therapeutic directions targeting glycan-binding motifs critical for brain development.

## Highlights

• Brain central synaptic organizers neurexins are heparan sulfate (HS) proteoglycans
• A dual HS and protein domain mode mediates neurexin binding to neuroligin and LRRTM
• HS increases neurexin heterogeneity and expands its interactome to HS binding factors
• HS glycan on neurexin is required for normal synaptic development and mouse survival

## Graphical Abstract

**Figure:**
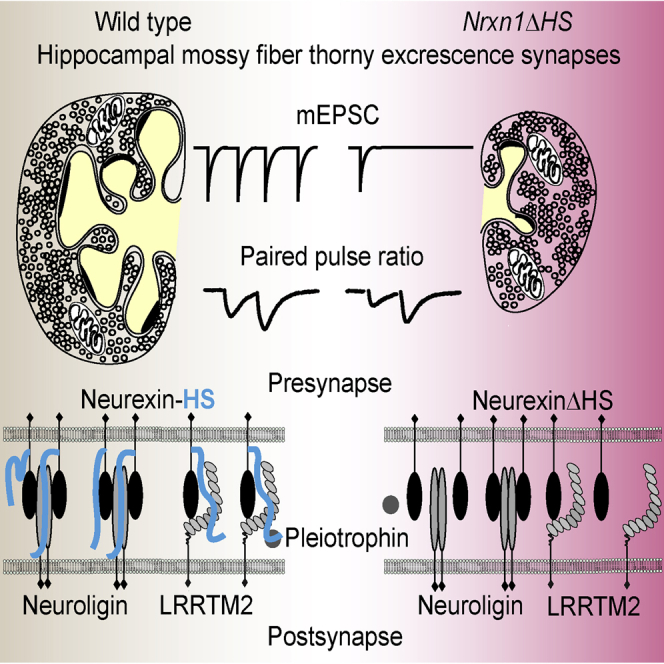

## Teaser

Neurexins, major synaptic-organizing proteins, are heparan sulfate (HS) proteoglycans, and HS modification is required for neurexin functions in synaptic transmission, development, and behavior.

## Introduction

Synaptic junctions as modifiable functional units play an essential role in information processing in the brain. Neurexin (Nrx) synaptic organizing proteins are thought to be central effectors of synaptic properties that shape the activity of neural circuits (Südhof, 2017). The most well-known ligand for Nrx is neuroligin (NL). Together, Nrx and NL recruit proteins to developing synapses and promote multiple aspects of synapse assembly, maturation, and plasticity (Krueger et al., 2012, Reissner et al., 2013, Südhof, 2017). *Nrxn* and *Nlgn* genes function in overlapping patterns in essentially all brain circuits and are necessary for mouse survival (Missler et al., 2003, Varoqueaux et al., 2006).

Considerable heterogeneity contributes to functional selectivity of different Nrx-NL complexes. Mammals have 3 Nrx genes, each of which uses two promoters to generate longer α and shorter β forms, and 6 sites of alternative splicing to generate altogether >1,500 forms (Südhof, 2017, Schreiner et al., 2014). There are 4 NL genes in mice and 5 in humans, also regulated by alternative splicing, with NL1 selective for excitatory glutamatergic and NL2 selective for inhibitory GABAergic and glycinergic synapses (Krueger et al., 2012). Nrx also acts through postsynaptic partners other than NLs, primarily LRRTM1 and LRRTM2 (Roppongi et al., 2017), as well as Cbln1-Gluδ2 in cerebellum (Uemura et al., 2010), interactions that are regulated by Nrx splicing.

These interactions of Nrx with postsynaptic ligands are thought to be mediated purely by protein domains. The peptide interaction mode between Nrx and NL is well accepted based on crystal structures and mutagenesis studies from multiple labs (Bourne and Marchot, 2014). The acetylcholinesterase-homology domain of each NL in a dimer binds the laminin neurexin sex hormone binding (LNS) domain common to α and β Nrx with KD values in the 10^−8^–10^−5^ M range.

There is strong evidence implicating altered Nrx and NL function in human psychiatric disorders. Autism-associated mutations in multiple human *NRXNs* and *NLGNs* are consistently found, including copy number variations, microdeletions, and truncating nonsense and function-altering missense mutations (Huguet et al., 2013, Südhof, 2017). Although rare (in <1% of patients), *NRXN1* mutations are also among the most frequent single-gene mutations in both schizophrenia (Rees et al., 2014) and Tourette’s Syndrome (Huang et al., 2017).

Heparan sulfate proteoglycans (HSPGs) are also implicated in synaptic function and autism, yet the molecular mechanisms remain uncertain. In addition to well-studied roles in brain development, HSPGs are implicated in activity-induced synaptic plasticity and regulation of oscillatory activity in mature brain networks (Farhy Tselnicker et al., 2014, Minge et al., 2017). *EXT1* and *HS3ST5*, which encode an essential HS biosynthetic enzyme and an HS sulfotransferase, respectively, have both been associated with autism susceptibility (Li et al., 2002, Wang et al., 2009). Late postnatal deletion of *Ext1* in a subset of neurons leads to deficits in synaptic function and autism-like social, communication, and stereotypy behaviors in mice (Irie et al., 2012) through unknown mechanisms.

Here, we shed light on the links between HSPG and Nrx pathways by establishing that Nrxs are themselves HSPGs. HS glycan modification of Nrx is critical for high-affinity interactions with NLs and LRRTMs, mediates interaction with additional ligands, and is essential for normal synapse development at *Drosophila* neuromuscular junctions and at mouse hippocampal mossy fiber CA3 synapses. Thus, HS is a critical component of Nrx, a component that further expands the diversity of Nrx structure and molecular interactions, providing a molecular basis for fine-tuning Nrx function.

## Results

### Nrxs Are Evolutionarily Conserved HSPGs

**Figure 1:**
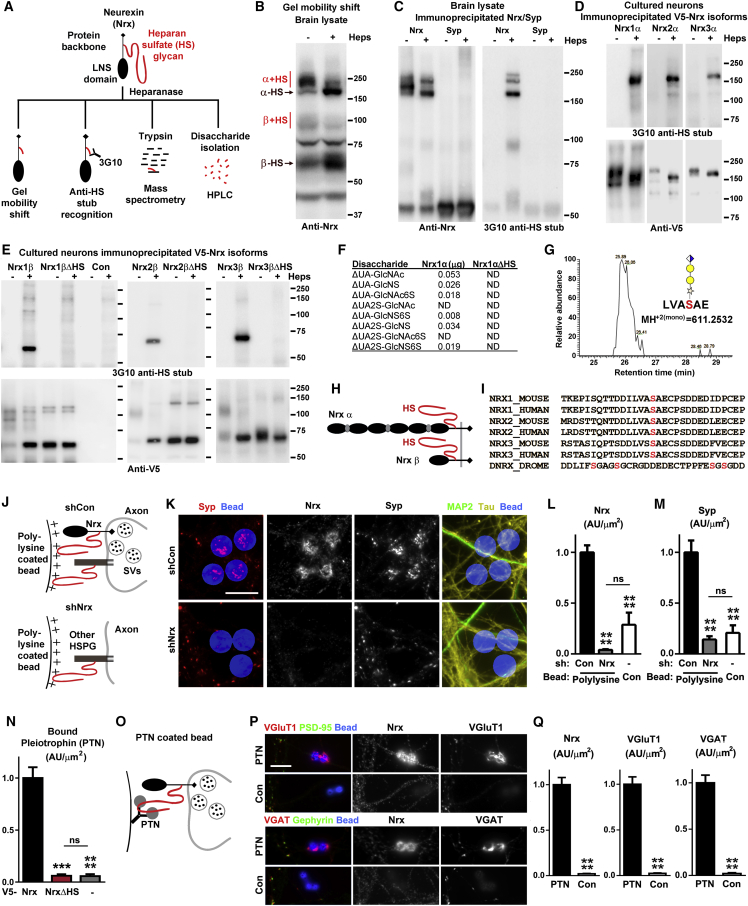
Nrxs Are HSPGs and Mediate Presynaptic Induction by HS Binding Factors (A) Schematic outline for identification of Nrxs as HSPGs. (B) Native Nrx shows molecular weight shifts upon cleavage of HS with heparinase (Heps). Prominent Nrx α and β bands are indicated. (C) Immunoprecipitated native Nrx shows molecular weight shifts with heparinase and is recognized by an HS antibody; 3G10 recognizes the HS stub remaining after heparinase cleavage (intact HS is not well recognized by western blot and the heparinase treatment helps condense the bands to improve detection). Anti-synaptophysin (syp) was used as a control for immunoprecipitation. (D) All recombinant V5-tagged α Nrx expressed in neurons show molecular weight shifts with heparinase and are recognized by an HS antibody. (E) Point mutation of each V5-tagged β Nrx lacking splice inserts confirms the HS modification site in neurons; Nrx1,2,3βΔHS Ser→Ala mutants lack HS modification; Con used empty vector. The untreated V5-Nrxβ signal may appear diffuse due to modification with HS chains of varying length; the signal collapses to one band upon heparinase treatment. These assays were done in neurons as Nrx is poorly modified in cell lines (Figure S1B). (F) HPLC following heparinase cleavage confirms the presence of HS disaccharides in purified Nrx1α but not Nrx1αΔHS ectodomain. ΔUA, Δ^4,5^-unsaturated uronic acid; GlcNAc, N-acetylglucosamine; GlcNS, N-sulfoglucosamine; 2S, 2-O-sulfation; 6S, 6-O-sulfation; ND, not detected. (G) Identification of HS modified peptide from Nrx1α recombinant protein. Selected Isotopic Chromatograph of m/z 611.2532 (10 ppm mass tolerance) corresponding to the glycopeptide ‘LVAS(Pentose-Hexose-Hexose-Uronic Acid)AE’ from NRx1α, which is consistent with the Xylose-Galactose-Galactose-Glucuronic Acid tetrasaccharide that would remain attached to the protein backbone of an HSPG following digestion with heparinase. The fragmentation ions from this glycopeptide were weak in intensity, most likely due to the poor modification of Nrx in HEK293 cells and the relative complexity of the starting material coupled with the necessity to remove all disaccharides to allow for ionization and detection by nanospray MS. No signal was detected within 20 ppm mass tolerance corresponding to ‘LVAS(Pentose-Hexose-Hexose-Uronic Acid)AE’ or ‘LVAA(Pentose-Hexose-Hexose-Uronic Acid)AE’ from NRx1αΔHS, consistent with this mutation abolishing HS modification. (H and I) The position of the conserved HS modification in Nrx is shown on the red serine in this sequence between the LNS and transmembrane domains, as identified in Figures S1C, S1E and in (E) and (G); for Dnrx, we did not distinguish whether all or a subset of these serines are modified. (J–M) Clustering of presynaptic marker synaptophysin (Syp), a component of synaptic vesicles (SVs), and of Nrx induced by polylysine-coated beads was reduced by Nrx knockdown (shNrx) in comparison to control (shCon) at or below levels associated with uncoated (Con) beads. Measures are integrated intensity of puncta per contact area of beads with tau-positive axons lacking contact with microtubule-associated protein 2 (MAP2)-positive dendrites to exclude native synapses (AU, arbitrary units). ^∗∗∗∗^p < 0.0001 by Kruskal-Wallis and Dunn’s tests compared to shCon polylysine beads, n = 18–33 cells or 16–30 beads from 2–3 independent experiments. (N) Recombinant pleiotrophin (PTN) bound to immature neurons expressing V5-Nrx, but not V5-NrxΔHS. Immature neurons were used because Nrx is not well modified in cell lines and the low levels of native Nrx were insufficient to mediate detectable binding to untransfected cells. ^∗∗∗^p < 0.001 and ^∗∗∗∗^p < 0.0001 by Kruskal-Wallis and Dunn’s tests compared to Nrx, n = 17–38 cells from 2 independent experiments. (O–Q) Beads coated with pleiotrophin induced clustering of native Nrx with vesicular glutamate transporter VGluT1 or with vesicular GABA transporter VGAT in contacting axons at sites lacking postsynaptic PSD-95 or gephyrin, respectively. Measures are integrated intensity of Nrx, VGluT1, or VGAT per bead area lacking postsynaptic markers. ^∗∗∗∗^p < 0.0001 by Mann-Whitney test, n = 27–33 beads from 2 independent experiments. Western blot results are representative of two (C) or three (B, D, E) biological replicates. Error bars represent SEM. Scale bars: (K) 10 μm, (P) 20 μm. See also Figures S1 and S2 and Tables S1 and S2.

**Figure S1:**
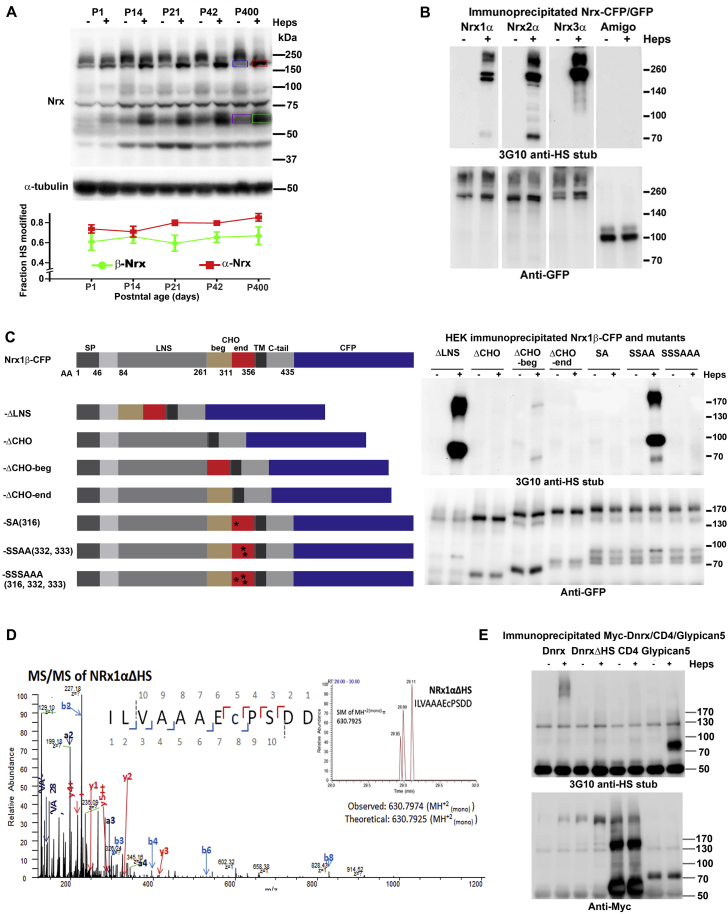
Nrxs Are HSPGs, Related to Figure 1 (A) Nrx HS modification throughout development. Crude synaptosome fraction was prepared from mouse brain at the postnatal ages indicated (in days, e.g., P1 is postnatal day 1) and treated or not with heparinase. The extent of Nrx HS modification at each age was estimated from the Nrx immunoblot. For example for P400, the fraction of α-Nrx lacking HS modification was estimated as the integrated signal of unmodified α-Nrx in the untreated sample (blue box) divided by total α-Nrx (red box, condensed band for unmodified α-Nrx after heparinase). Similarly, for P400 the fraction of β-Nrx lacking HS modification was estimated as the integrated signal in the purple box divided by the integrated signal in the green box. In the graph shown, values are reported as fraction of α-Nrx or β-Nrx bearing HS modification. Two-way ANOVA revealed a significant effect of postnatal age (p < 0.05) but posthoc Bonferroni’s pairwise comparisons were not significant, n = 3. Error bars represent SEM. (B) Nrx is poorly HS modified in HEK293 cells in comparison with neurons. All recombinant Nrx fused with GFP or CFP expressed in HEK293 cells are recognized by an HS antibody, indicating some degree of HS modification; 3G10 recognizes the HS stub remaining after heparinase cleavage. However, heparinase treatment resulted in little or no shift in molecular weight, in contrast with the apparent molecular weight shifts of Nrx from the brain or primary neurons (Figures 1A–1C), indicating a deficient modification level in HEK293 cells. (C) Mapping the HS modification site. SP, signal peptide; LNS, laminin neurexin sex hormone binding protein domain; CHO, glycosylated region as previously reported (Reissner et al., 2013); CHO-beg, N-terminal half of the glycosylated region; CHO-end, C-terminal half of the glycosylated region; TM, transmembrane domain; C-tail, cytoplasmic domain; CFP, cyan fluorescent protein; ^∗^, serine to alanine point mutation. The Nrx1β lacked splice inserts. The constructs were expressed in HEK293 cells and lysate immunoprecipitated with anti-GFP antibody, treated or not with heparinase, then immunoblotted. 3G10 recognizes the HS stub remaining after heparinase treatment. Deletion of the glycosylated region or its C-terminal half or point mutation of serine 316 within this region to alanine (SA(316)) abolished HS modification, while deletion of the N-terminal half of the glycosylated region reduced but did not abolish HS modification. (D) Identification of unmodified peptide from Nrx1αΔHS recombinant protein. MS/MS of peptide ‘ILVAAAEcPSDD’ detected in Nrx1αΔHS. A Selected Ion Monitoring Chromatograph (SIM) of the whole mass at m/z MH+2(mono) 630.7925 with 10ppm mass tolerance is seen in the insert. Fragmentation was produced by Higher-energy collisional dissociation (HCD), resulting in y (red), b (light blue) ions, as well as a ions and internal ions (dark blue) necessary for confirmation of peptide sequence. Lower case ‘c’ refers to carbamidomethylated cysteine. This peptide was only found in its non-glycosylated form and only in the sample NRx1αΔHS. Results from the LC-MS/MS following the Glu-C and Heparinase I-III digestion showed 78% and 58% coverage of Nrx1α and Nrx1αΔHS, respectively. (E) *Drosophila* neurexin Dnrx is HS modified. Myc-tagged Dnrx and ΔHS mutant (LIFSGAGSGCRGDDEDECTPPFESGSGDD to LIFAGAGAGCRGDDEDECTPPFEAGAGDD) along with CD4 negative control and glypican-5 positive control were expressed in HEK293 cells. Lysate was immunoprecipitated with anti-Myc antibody, treated or not with heparinase, then immunoblotted. 3G10 recognizes the HS stub remaining after heparinase treatment.

We demonstrate by multiple lines of evidence that Nrxs are HSPGs (Figure 1A), joining a superfamily of 17 known HSPGs (Sarrazin et al., 2011, Xu and Esko, 2014). First, brain immunoblots for Nrx show molecular weight shifts upon cleavage of HS chains with heparinases I, II, and III (Figure 1B). HS chains contribute 20–100 kDa, with a heterogeneous chain length and structure even for a single-protein backbone. We estimate that approximately 80% of α Nrxs and 66% of β Nrxs are HS modified in adult mouse brain, and this fraction changes little throughout development (Figure S1A). Second, native brain Nrx and recombinant Nrx-1,2,3 α and β are all recognized by the anti-HS antibody 3G10, which recognizes the HS stub remaining after heparinase treatment (David et al., 1992) and recognized all heparinase-treated immunoprecipitated Nrx (Figures 1C–1E). Using this assay on Nrx1β deletion and point mutants lacking splice inserts, we mapped the HS modification site (Figure S1C; confirmed in Nrx-1,2,3 β in cultured neurons, Figure 1E). Third, purified recombinant Nrx1α ectodomain analyzed by strong anion exchange HPLC following heparinase treatment confirmed multiple HS disaccharides, which were not present for the Ser→Ala point mutant of the HS modified residue, Nrx1αΔHS (Figure 1F). Further, liquid chromatography-mass spectrometry (LC-MS) following protease and heparinase digestion revealed a molecular ion mass corresponding to the Nrx1α modified peptide bearing the HS stub (Figure 1G). This signal was detected only in Nrx1α, and not in Nrx1αΔHS. Conversely, unmodified peptide lacking glycosylation at this position was detected only in Nrx1αΔHS, and not Nrx1α (Figure S1D). Thus, four independent methods support the conclusion that all Nrxs are HSPGs (Figure 1A).

The HS modification site is conserved in all vertebrate Nrx genes from zebrafish to human (Figures 1H and 1I and Table S1). Although the primary sequence is more divergent in invertebrates, the single *Drosophila* neurexin (Dnrx) is HS modified in the same region between the last LNS and transmembrane domains (Figures 1H, 1I, and S1E). *Caenorhabditis elegans* neurexin also has a consensus potential HS modification site in this region (Table S1).

### HS Modification of Nrx Mediates Presynaptic Differentiation by Polylysine Beads and Novel Ligands

**Figure S2:**
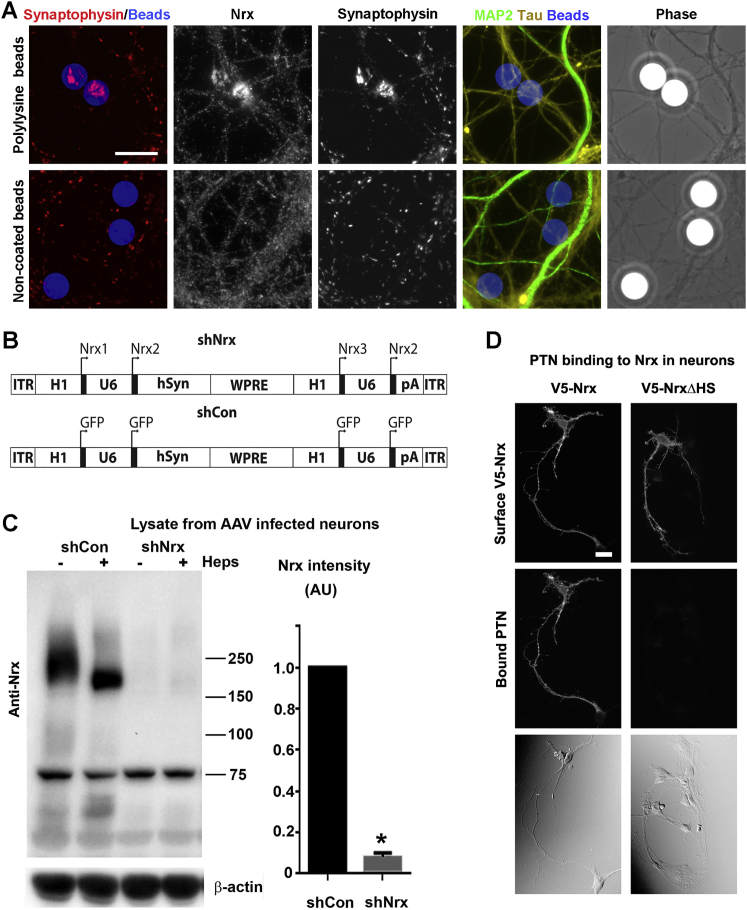
Polylysine-Coated Beads Induce Presynaptic Differentiation through Nrx, and Pleiotrophin Binds Nrx, Related to Figure 1 (A) Polylysine-coated beads but not uncoated beads induced clustering of Nrx and of presynaptic marker synaptophysin at contact sites with tau-positive axons; see Figures 1L and 1M for quantitation. Regions of MAP2-positive dendrite contact were excluded from the quantitation to exclude native synapses. For the experiments here and in Figures 1K–1M, beads were added to neuron cultures at 13 DIV and analysis performed at 15 DIV. Scale bar, 10 μm. (B) The components of the DNA packaged between the inverted terminal repeats (ITRs) in the AAV vector are shown. shNrx consisted of a cassette of shRNAs against rat Nrx1, Nrx2 (duplicated) and Nrx3, regions common to all isoforms, driven by H1 or U6 promoters, plus a human synapsin promoter (hSyn) and Woodchuck Hepatitis Virus posttranscriptional regulatory element (WPRE; we did not test whether the hSyn and WPRE are necessary). In shCon, the Nrx shRNAs were all replaced by shRNA against GFP. (C) Western blot of lysate from cultured rat hippocampal neurons exposed from 3 to 14 DIV with shCon or shNrx AAV. Parts of the samples were treated with heparinase to condense the Nrx bands. The band just above 75 kDa was presumed to be non-specific. Estimated knockdown efficiency of Nrx α and β combined was 92.1%; ^∗^p < 0.0001, t test, n = 3. Error bars represent SEM. (D) Recombinant pleiotrophin (PTN) bound to immature neurons expressing V5-Nrx but not V5-NrxΔHS; see Figure 1N for quantitation. Immature neurons were used because Nrx is not well modified in cell lines and the low levels of native Nrx were insufficient to mediate detectable binding to untransfected cells. Scale bar, 20 μm.

Polylysine-coated beads, long thought to non-specifically induce presynaptic differentiation (Burry, 1980), were recently found to do so by aggregating axonal HSPGs (Lucido et al., 2009). Syndecan-2 was aggregated and was suggested to be the HSPG responsible for polylysine-bead-induced presynaptic differentiation, but functional evidence was lacking. We find that polylysine beads aggregate axonal Nrxs (Figures 1J–1L and S2A). Furthermore, efficient knockdown of all Nrxs with an adeno-associated viral vector expressing multiple short hairpin RNAs (Figures S2B and S2C) revealed that Nrxs are required for polylysine-bead-induced presynaptic differentiation (Figures 1J–1M). Thus, these results suggest that polylysine-coated beads directly bind the HS chain of Nrx, and the recruited Nrx triggers local presynaptic assembly.

HS modification of Nrxs may also mediate interaction with native HS-binding proteins. Indeed, the HS-binding extracellular matrix proteins F-spondin and thrombospondin, a glial-derived factor that promotes synaptic differentiation (Christopherson et al., 2005), were recently found to bind Nrx (Traunmüller et al., 2016). We chose the HS-binding growth factor pleiotrophin (also known as heparin-binding growth-associated molecule), which shows activity-regulated expression in hippocampus (Lauri et al., 1996), to test as a novel candidate for Nrx interaction and synaptic function. Recombinant pleiotrophin bound to cell-expressed Nrx1β, but not Nrx1βΔHS (Figures 1N and S2D). Furthermore, pleiotrophin conjugated to inert beads aggregated axonal Nrxs and induced excitatory and inhibitory presynaptic differentiation (Figures 1O–1Q). Pleiotrophin, as a factor that interacts solely with HS chains of Nrx independent of its protein domains, may exemplify a new category of synapse regulatory factors. This new mode of interaction via HS chains increases the number of potential ligands for Nrx to several hundred (Table S2; Ori et al., 2011, Xu and Esko, 2014), complementing the roughly two dozen ligands for Nrx protein domains (Südhof, 2017).

### HS Modification of Nrx Is Required for Development of Functional Synapses in Cultured Hippocampal Neurons

**Figure 2:**
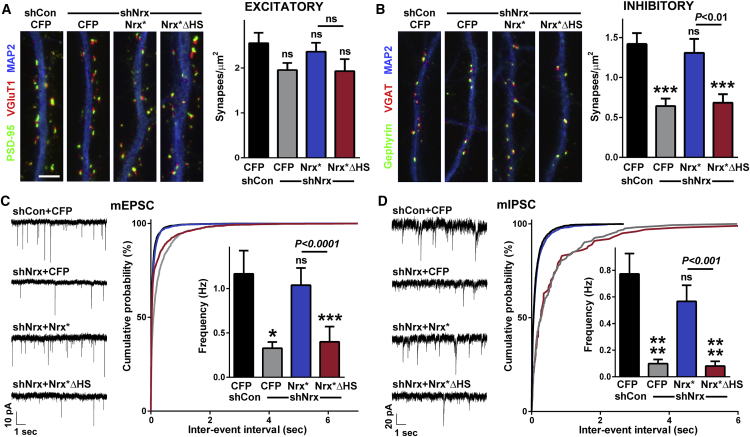
Nrx HS Modification Is Required for Development of Functional Synapses in Cultured Hippocampal Neurons (A) Density of excitatory synapses (apposed PSD-95-VGluT1 puncta) was not significantly altered by Nrx knockdown. (B) Density of inhibitory synapses (apposed gephyrin-VGAT puncta) was reduced by Nrx knockdown and rescued by RNAi-resistant Nrx^∗^ but not Nrx^∗^ΔHS. (C and D) Excitatory and inhibitory transmission were impaired by Nrx knockdown and rescued by RNAi-resistant Nrx^∗^, but not Nrx^∗^ΔHS. All inter-event interval distributions were significantly different except that shCon+CFP versus shNrx+Nrx^∗^, and shNrx+CFP versus shNrx+Nrx^∗^ΔHS, did not differ for mIPSCs; comparing Nrx^∗^ΔHS with Nrx^∗^ rescue gave p < 0.05 for mEPSCs and p < 0.001 for mIPSCs by Kruskal-Wallis and Dunn’s tests. Amplitude data are shown in Figure S3C. ^∗^p < 0.05, ^∗∗∗^p < 0.001, and ^∗∗∗∗^p < 0.0001 by Kruskal-Wallis and Dunn’s tests compared to shCon+CFP; n = 39–55 (A and B), 21–32 (C), or 18–20 (D) from 3–4 independent experiments. shNrx plus Nrx^∗^ΔHS not significant compared with shNrx plus CFP for all assays. Error bars represent SEM. Scale bars: 5 μm. See also Figure S3.

**Figure S3:**
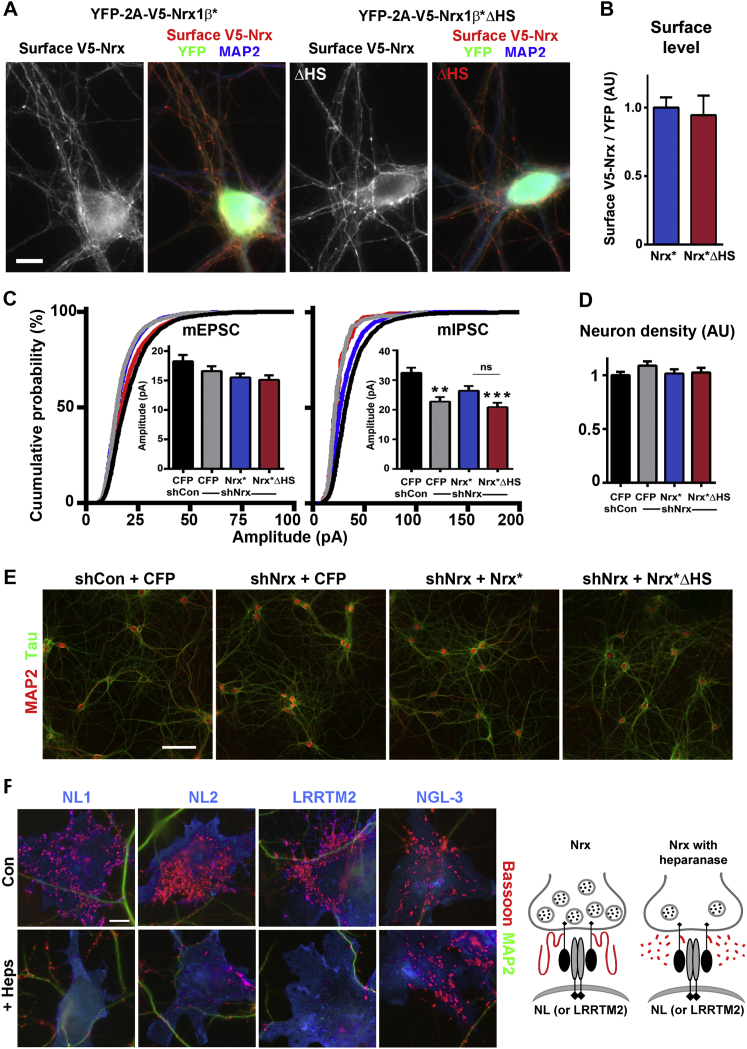
Nrx HS Modification Does Not Affect Nrx Surface Trafficking or Neuron Survival, and Heparinase Reduces Presynaptic Differentiation, Related to Figures 2 and 3 (A) Nrx1β^∗^ΔHS reaches the axon surface, like Nrx1β^∗^, as shown by surface labeling of the V5 tagged Nrx constructs co-expressed with YFP through a 2A peptide. Note that both Nrx1β^∗^ and the ΔHS mutant are primarily axonal as MAP2 positive dendrites show little V5 fluorescence. (B) The axon surface level of Nrx1β^∗^ΔHS was indistinguishable from that of Nrx1β^∗^. Ratios of surface V5 to total YFP were assessed along axons of 13 DIV neurons co-expressing the V5 tagged Nrx constructs with YFP linked through the 2A peptide. A Mann-Whitney test showed no significant difference, n = 21-22 cells from 2 independent experiments. (C) mEPSC amplitude was not significantly different among groups (p = 0.0591). The numbers of cells were: CFP+shCon (n = 21); CFP+shNrx (n = 32); Nrx^∗^+shNrx (n = 29); Nrx^∗^ΔHS+shNrx (n = 32) from 4 independent experiments. mIPSC amplitude was reduced by Nrx knockdown and partially rescued by RNAi-resistant Nrx^∗^ but not Nrx^∗^ΔHS. The numbers of cells were: CFP+shCon (n = 20); CFP+shNrx (n = 18); Nrx^∗^+shNrx (n = 18); Nrx^∗^ΔHS+shNrx (n = 18) from 3 independent experiments. ^∗∗^p < 0.01 and ^∗∗∗^p < 0.001 by Kruskal-Wallis and Dunn’s tests compared to shCon+CFP. For the experiments shown here and in Figure 2, neurons were transfected at plating with the rescue constructs, exposed to knockdown AAV vectors from 3 DIV and analyzed at 13 DIV (mIPSCs) or 14 DIV (mEPSCs). (D and E) Hippocampal neuron cultures immunolabeled for MAP2-positive dendrites and tau-positive axons appeared similar among all groups. Neuron density assessed as number of MAP2-positive neurons per coverslip area normalized to the shCon+CFP group did not differ among groups by Kruskal-Wallis, n = 30 from 3 independent experiments. Neurons analyzed here were from the same experiments as for Figures 2, 3D-3E and panel C in this figure. Neurons were transfected at plating with the rescue constructs, exposed to knockdown AAV vectors from 3 DIV and analyzed at 14 DIV. (F) Clustering of presynaptic marker bassoon in contacting axons induced by NL1, NL2, or LRRTM2 on COS7 cells, but not by NGL-3 a ligand of type IIA protein tyrosine phosphatases, was reduced by heparinase (Heps). Co-labeling for the microtubule associated protein 2 (MAP2) identified dendrites to exclude native synapses from the analysis. Heparinase was added after axon outgrowth only during the co-culture period. For quantitation, see Figure 3C. Error bars represent SEM. Scale bars, (A) 10 μm, (E) 10 μm, (F) 100 μm.

To test the role of Nrx HS modification in native synaptic development and function, we used a molecular replacement strategy in cultured hippocampal neurons, knocking down all Nrx and rescuing with a mixture of RNAi-resistant Nrx 1α and 1β wild-type (Nrx^∗^) or point-mutant-abolishing HS modification (Nrx^∗^ΔHS; Figure 2; confirmed to reach the axon surface, Figures S3A and S3B). Excitatory synapse density, assessed as clusters of postsynaptic scaffold apposed to presynaptic vesicles, was not significantly altered (Figure 2A), but inhibitory synapse density was reduced by Nrx knockdown (Figure 2B), consistent with the phenotype of α-Nrx or global Nrx mutant neurons (Chen et al., 2017, Missler et al., 2003). Inhibitory synapse density was fully rescued by expression of Nrx^∗^, whereas Nrx^∗^ΔHS showed no rescue. While loss of α-Nrx does not affect excitatory synapse density, it dramatically reduces vesicle fusion and excitatory and inhibitory synaptic transmission (Missler et al., 2003); thus, we assessed synapse function. Nrx knockdown resulted in markedly reduced frequency of miniature excitatory and inhibitory postsynaptic currents (mEPSCs and mIPSCs; Figures 2C and 2D). Furthermore, the reduction in both mEPSC and mIPSC frequency was restored in Nrx-knockdown cultures by expression of Nrx^∗^, whereas Nrx^∗^ΔHS mediated no functional rescue. Neuron density did not differ among groups (Figures S3D and S3E). Thus, HS modification of Nrx is essential for its functions in excitatory and inhibitory synaptic transmission and morphological inhibitory synapse development in cultured hippocampal neurons.

### HS Modification of Nrx is Required for Presynaptic Differentiation by Nrx Ligands NLs and LRRTMs

**Figure 3:**
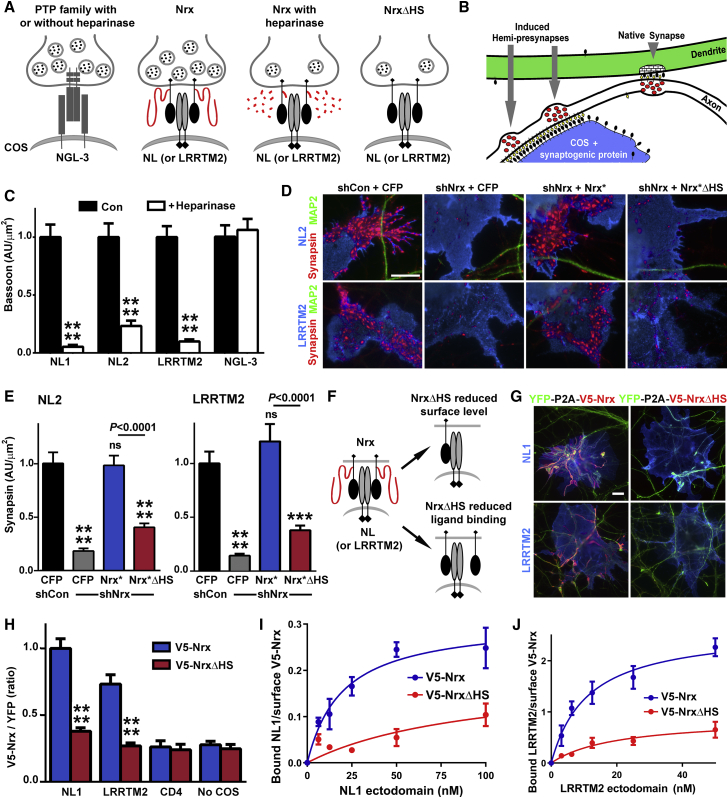
Nrx HS Modification Is Required for Presynaptic Differentiation by NLs and LRRTM2 (A and B) Schematic models for panels (C)–(E). (C) Clustering of presynaptic marker bassoon in contacting axons induced by Nrx ligands NL1, NL2, or LRRTM2 on COS7 cells, but not by NGL-3 a ligand of type IIA protein tyrosine phosphatases, was reduced by heparinase. Heparinase was added after axon outgrowth only during the co-culture period. Measures are integrated intensity of bassoon puncta, lacking dendrite contact to exclude native synapses, per transfected COS7 cell contact area. (D and E) Clustering of presynaptic marker synapsin induced by NL2 or LRRTM2 was abolished by Nrx knockdown (shNrx) and rescued by RNAi-resistant Nrx^∗^ but not Nrx^∗^ΔHS. Measures are integrated intensity of synapsin puncta per transfected COS7 cell-axon contact area lacking MAP2 dendrite contact. (F) Potential mechanisms by which Nrx HS modification might control synapse development. (G and H) HS modification of Nrx regulates its recruitment by NL1 and LRRTM2. Neurons were transfected for YFP-P2A-V5-Nrx or YFP-P2A-V5-NrxΔHS and co-cultured with COS7 cells expressing NL1, LRRTM2, or control CD4. Measures are integrated intensity of V5-Nrx or V5-NrxΔHS per contact area of YFP-positive axons with transfected COS7 cells lacking dendrite contact (no COS7 indicates intensity on YFP-positive axons not contacting COS7 cells). (I and J) HS modification of Nrx regulates interaction with NL1 and LRRTM2. Binding of NL1 or LRRTM2 ectodomain was measured on immature neurons expressing V5-Nrx or V5-NrxΔHS. For NL1 (I), scatchard analysis of this cell-based binding revealed a significant difference (p < 0.0001) with apparent Kd 19.7 and Bmax 0.31 for Nrx and Kd 95.2 and Bmax 0.19 for NrxΔHS. For LRRTM2 (J), scatchard analysis revealed a significant difference (p < 0.0001) with apparent Kd 11.1 and Bmax 2.6 for Nrx and Kd 19.8 and Bmax 0.9 for NrxΔHS. ^∗∗∗^p < 0.001 and ^∗∗∗∗^p < 0.0001 by Kruskal-Wallis and Dunn’s tests comparing each heparinase with Con (C, n = 28–33) or compared to shCon+CFP (E, n = 39–55) or comparing each V5-NrxΔHS to V5-Nrx (H, n = 40–57). Error bars represent SEM. Scale bars: 10 μm. See also Figure S4.

**Figure S4:**
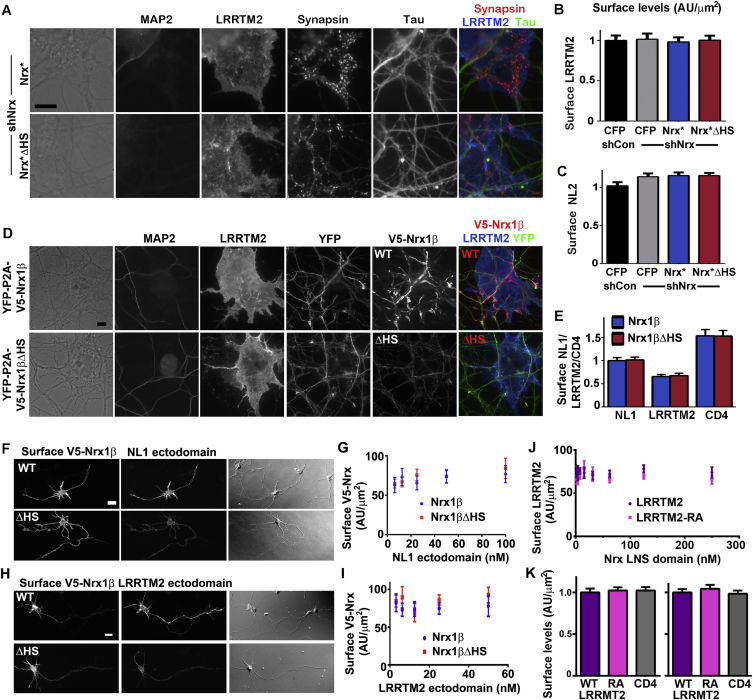
Nrx HS Modification Is Required for the Function of NL and LRRTM2, Related to Figures 3 and 4 (A) Clustering of presynaptic marker synapsin induced by LRRTM2 in co-culture was abolished by Nrx knockdown and rescued by RNAi-resistant Nrx^∗^ but not Nrx^∗^ΔHS. All single channel images are shown here corresponding to the fields in Figure 3D. (B and C) Surface levels of LRRTM2 (B) or NL2 (C) on the COS7 cells did not differ among Nrx knockdown and rescue groups; see Figure 3E for synapsin quantification. Neurons were transfected at plating with the rescue constructs, exposed to knockdown AAV vectors from DIV 3 and co-culture performed at DIV 13-14. (D and E) HS modification of Nrx regulates its recruitment by NL1 or LRRTM2. Shown here (D) are all single channel images for LRRTM2 corresponding to the fields in Figure 3G; see Figure 3H for Nrx quantification. Surface levels of NL1, LRRTM2 or CD4 in the COS7 cells did not differ among groups in this assay for recruitment of V5-Nrx1β or the ΔHS mutant (E). (F and G) Recombinant NL1 ectodomain bound more strongly to V5-Nrx1β compared to V5-Nrx1βΔHS expressed on the surface of neurons at 3 DIV (F); see Figure 3I for quantification. The level of surface expression of Nrx did not differ among all groups at the different concentrations of ligand (G). (H and I) Recombinant LRRTM2 ectodomain bound more strongly to V5-Nrx1β compared to V5-Nrx1βΔHS expressed on the surface of neurons at 3 DIV (H); see Figure 3J for quantification. The level of surface expression of Nrx did not differ among all groups at the different concentrations of ligand (I). (J) Surface expression of LRRTM2 in the Nrx LNS ectodomain binding experiment shown in Figure 4E did not differ between wild-type and the RA mutant at the different concentrations of ligands. (K) Surface expression of LRRTM2 in the co-culture experiment shown in Figures 4F–4I did not differ between wild-type and the RA mutant. The left graph corresponds to the experiment in Figure 4G whereas the right graph corresponds to Figure 4I. Error bars represent SEM. Scale bar: (A and D) 10 μm, (F and H) 20 μm.

Nrxs organize synapses primarily through interactions with postsynaptic NLs and LRRTMs, as well as cerebellar Cbln1-Gluδ2 (Krueger et al., 2012, Reissner et al., 2013, Roppongi et al., 2017). Thus, to understand mechanistically why HS modification of Nrx is important for synaptic development and function, we explored links between NLs and LRRTMs and the HS chain of Nrx. We first tested whether HS is required for NL1, NL2, or LRRTM2 to induce presynaptic differentiation. Expression of these Nrx partners on COS7 cells followed by co-culture with neurons triggers local aggregation of Nrx on the axon surface at points of COS7 cell contact, thereby inducing local presynaptic differentiation (Figures 3A and 3B). Acute removal of HS with heparinase abolished the ability of NL1, NL2, and LRRTM2 to induce presynaptic differentiation (Figures 3C and S3F). Heparinase did not affect presynaptic induction by NGL-3, which acts through an Nrx-independent parallel pathway involving type IIA protein tyrosine phosphatases (Takahashi and Craig, 2013). Furthermore, presynaptic induction by NL2 and LRRTM2 was abolished by Nrx knockdown and rescued by co-expression of Nrx^∗^, but not Nrx^∗^ΔHS (Figures 3D, 3E, and S4A–S4C).

Two simple mechanisms (Figure 3F) could explain why HS modification of Nrx is essential for Nrx ligand-induced presynaptic differentiation: (i) HS may stabilize Nrx on the axon surface, or (ii) HS may enhance ligand binding. To test the first possibility, we assessed surface expression of recombinant Nrx but found no difference between Nrx1β and Nrx1βΔHS (Figures S3A and S3B). To test the second possibility, whether HS modification affects the interaction between Nrx and its ligands, we did cell-based recruitment and binding assays. Recombinant Nrx1β expressed in neurons was recruited to contact sites by NL1 or LRRTM2 on COS7 cells, whereas Nrx1βΔHS was poorly recruited (Figures 3G, 3H, S4D, and S4E). Moreover, purified NL1 ectodomain and LRRTM2 ectodomain each showed greater binding to axonal surface-expressed Nrx1β relative to Nrx1βΔHS (Figures 3I, 3J, and S4F–S4I). These findings suggest that HS modification of Nrx in neurons increases its interaction with both NL and LRRTM ligands.

### Binding of Nrx Ligand LRRTM2 to HS Is Required for its Role in Presynaptic Differentiation

**Figure 4:**
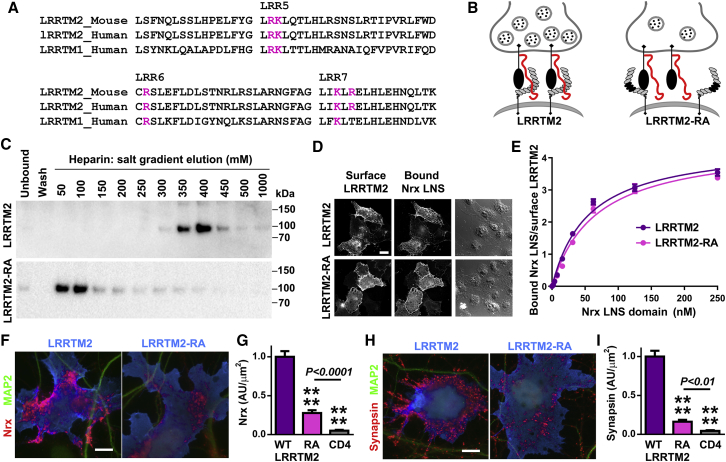
LRRTM2 Binding to HS Is Required for its Role in Presynaptic Differentiation (A) The proposed HS binding region in LRRTM is shown, with residues in pink mutated to Ala in LRRTM2-RA. (B) Schematic model. LRRTM2 binds to protein and HS domains of Nrx; reduction of HS binding by the LRRTM2-RA mutation may result in maintenance of some LRRTM2-Nrx complexes by the protein domain interactions and disruption of other complexes. (C) LRRTM2 ectodomain binds heparin, and binding is reduced by the RA mutation. Elution at higher salt indicates stronger binding. (D and E) LRRTM2 RA mutation does not affect binding to the Nrx LNS domain. Binding of Nrx LNS domain was measured on COS7 cells expressing LRRTM2 or LRRTM2-RA. Scatchard analysis of this cell-based binding revealed no significant difference (p > 0.1). (F–I) LRRTM2-RA is deficient at inducing presynaptic differentiation. Clustering of native Nrx (F and G) and synapsin (H and I) in contacting axons induced by LRRTM2 on COS7 cells was impaired by the RA mutation. Measures are integrated intensity of Nrx or synapsin puncta per transfected COS7 cell-axon contact area lacking MAP2 dendrite contact. ^∗∗∗∗^p < 0.0001 by Kruskal-Wallis and Dunn’s tests compared to wild-type LRRTM2 (G, n = 43–48 and I, n = 31–33) from 3-4 independent experiments. Surface levels of LRRTM2 and LRRTM2-RA did not vary in these co-culture assays (Figure S4). Error bars represent SEM. Scale bars: 20 μm. See also Figure S4.

Based on the above results and considering the previous finding that the related LRRTM4 binds HS (de Wit et al., 2013, Siddiqui et al., 2013), we propose that LRRTM2 directly binds HS. We propose that the binding site involves a series of basic residues aligned in leucine-rich repeats 5–7 (Figure 4A) and that this interaction, together with the binding of a region involving leucine-rich repeat 9 to the Nrx LNS domain (Siddiqui et al., 2010), mediates high-affinity interaction of LRRTM2 with Nrx (Figure 4B). In support of this hypothesis, recombinant LRRTM2 ectodomain bound strongly to a heparin column, and binding was diminished by mutating five Arg or Lys (pink in Figure 4A) to Ala in LRRTM2-RA (Figure 4C). This RA mutation did not affect binding of LRRTM2 to the Nrx LNS domain (Figures 4D, 4E, and S4J), thus specifically disrupting heparin/HS interaction. We next tested the significance of HS binding for the synaptogenic activity of LRRTM2 using the co-culture assay. While LRRTM2-RA showed some synaptogenic activity, consistent with its binding to the Nrx LNS domain, its ability to recruit native Nrx on contacting neurons and to induce presynaptic differentiation was greatly impaired (Figures 4F–4I and S4K). Thus, LRRTM2 requires HS binding for full Nrx interaction and presynaptic differentiation.

### Binding of Nrx Ligand NL to HS Is Required for its Role in Synapse Development

The major role of Nrx HS modification in synaptic development, particularly for inhibitory synapses (Figures 2B and 2D), is unlikely to be mediated solely through LRRTMs, which localize specifically to excitatory synapses, where they cooperate functionally with NLs (Roppongi et al., 2017). Moreover, although it initially seemed unlikely to us that LRRTMs and NLs independently evolved binding sites for HS, as well as for the Nrx LNS domain, our interaction data (Figures 3G–3J) supported this possibility. Thus, we considered the hypothesis that NLs, the best-characterized evolutionarily conserved *trans*-synaptic partners of Nrx (Krueger et al., 2012), directly bind HS.

**Figure 5:**
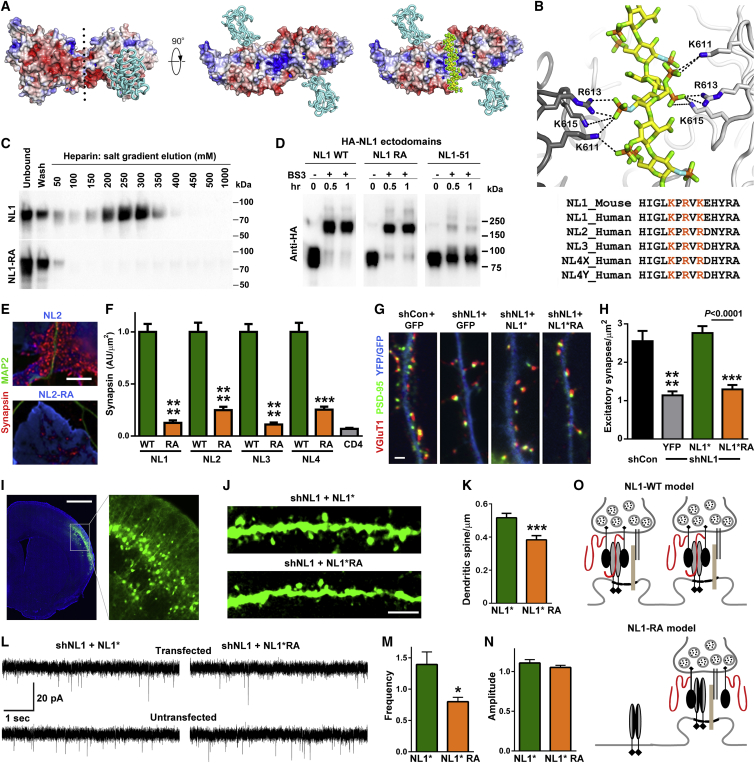
NL Binding to HS Is Required for its Synapse Promoting Activity (A) The NL1-Nrx1β LNS domain complex (PDB: 3VKF), side view. The NL1 surface is colored according to the electrostatic potential from red (−8 k_b_T/e_c_) to blue (+8 k_b_T/e_c_), the Nrx LNS domain is in aquamarine cartoon representation. Dotted line in the left panel indicates the 2-fold pseudo-symmetry axis of a NL1 dimer. The middle panel is rotated 90 degrees, corresponding to a view from the presynaptic side. This reveals a large basic surface lining the canyon formed between two NL1 molecules. A heparin dodecamer (PDB: 1HPN, shown in sphere representation, carbon atoms yellow; oxygen chartreuse) matches perfectly the dimensions of this canyon. Fitting was done manually. (B) Close-up of possible interactions between docked heparin and basic residues lining the NL1 canyon (top), indicating the Arg or Lys residues mutated in this study and highlighted in orange in the sequences (bottom). Black dotted lines indicate putative hydrogen bonds. (C) The NL1 ectodomain binds heparin, and binding is abolished by the RA mutation. (D) The NL1 RA mutation does not interfere with its dimerization. Purified recombinant HA-tagged ectodomains of NL1 wild-type, RA mutant, and the −51 mutant reported to disrupt dimerization (Ko et al., 2009) were chemically cross-linked by treatment with 0.5 mM bis (sulfosuccinimidyl) suberate-d_0_ (BS3) for the indicated amounts of time. (E and F) Presynaptic differentiation in contacting axons induced by each NL on COS7 cells was impaired by the RA mutations. ^∗∗∗^p < 0.001 and ^∗∗∗∗^p < 0.0001 by Kruskal-Wallis and Dunn’s tests comparing NL RA with WT, n = 24–36 cells from 3 independent experiments. NL RA mutants did not differ significantly from CD4 control. Surface levels of NLs did not vary (Figure S5). (G and H) Density of excitatory synapses (apposed PSD-95-VGluT1 puncta) in cultured hippocampal neurons was reduced by NL1 knockdown and rescued by RNAi-resistant NL1^∗^ but not NL1^∗^-RA. ^∗∗∗^p < 0.001 and ^∗∗∗∗^p < 0.0001 by Kruskal-Wallis and Dunn’s tests, n = 31–39 cells from 3 independent experiments. (I) NL1 knockdown and replacement with NL1^∗^ or NL1^∗^-RA plus GFP in mouse cortex by in utero electroporation. (J and K) Spine density in layer 2/3 cortical neurons was reduced in NL1 knockdown cells expressing NL1^∗^-RA compared with NL1^∗^. ^∗∗∗^p < 0.001 by Mann-Whitney test, n = 33–34 dendrites from 3 mice each. (L-N) mEPSCs were recorded from neighboring transfected and untransfected layer 2/3 cortical neurons. Data are presented as the ratio of average frequency or amplitude for transfected over untransfected cells. The NL1^∗^-RA group showed a reduction in frequency and amplitude compared with the NL1^∗^ group. ^∗^p < 0.05 by t test, n = 5–6 mice averaging from 2–9 cells per mouse. Individual cell data are shown in Figure S5. (O) Model for NL1-containing synapses. NL1 binds to protein and HS domains of Nrx. Loss of HS binding by the NL1-RA mutation may result in loss of some synapses and maintenance of other synapses through NL1-Nrx protein domain interactions and additional synaptic organizing complexes. Error bars represent SEM. Scale bar: (E) 10 μm, (G) 2 μm, (I) 1 mm, (J) 5 μm. See also Figure S5.

**Figure S5:**
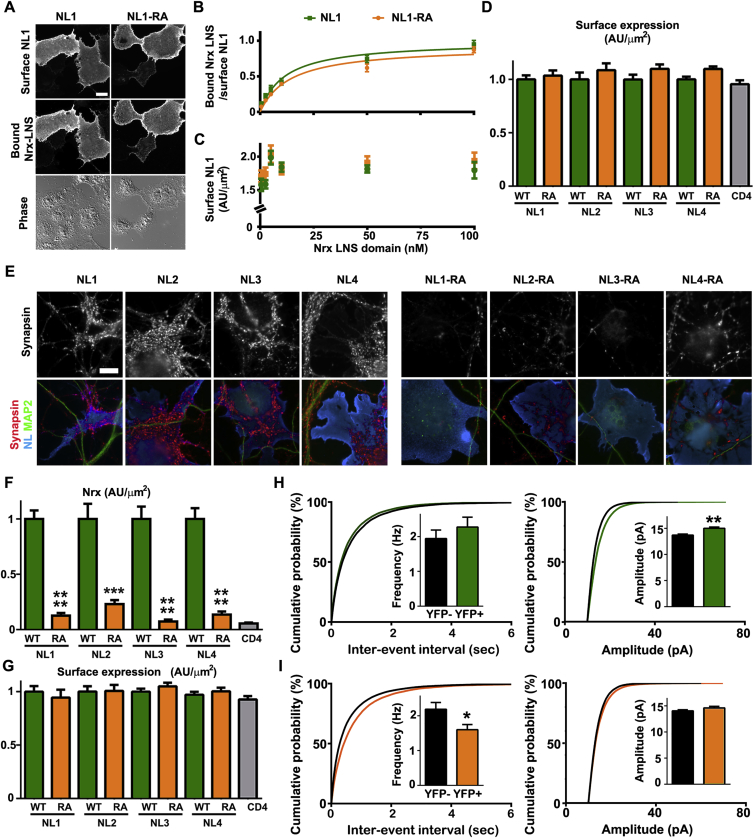
NLs and LRRTM2 Bind to HS for Synaptic Development, Related to Figure 5 (A-C) The RA mutations on NL1 do not affect its binding to the Nrx LNS domain. Recombinant Nrx LNS ectodomain bound to NL1-RA as well as to wild-type NL1 expressed on the surface of COS7 cells (A). Scatchard analysis of this cell-based binding revealed no significant difference (p > 0.1; B). The level of surface expression of NL1 did not differ among all groups at the different concentrations of ligand (C). (D and E) RA mutation of NLs 1-4 impairs their ability to induce presynaptic differentiation. In the co-culture assay, clustering of presynaptic marker synapsin in contacting axons was induced well by all NL wild-type on COS7 cells (left two columns), but poorly by the RA mutants (right two columns); see Figure 5F for quantification. Surface expression of NLs in the co-culture experiment did not differ between wild-type and the RA mutant (E). (F and G) Recruitment of native Nrx in contacting axons induced by each NL on COS7 cells was impaired by the RA mutations. ^∗∗∗^p < 0.001 and ^∗∗∗∗^p < 0.0001 by Kruskal-Wallis and Dunn’s tests comparing NL RA with WT, n = 28-37 cells from 3 independent experiments. NL RA mutants did not differ significantly from CD4 control. Surface expression of NLs in the co-culture experiment did not differ between wild-type and the RA mutant (G). (H and I) The raw mEPSC data from Figures 5M and 5N is presented here. ^∗^p < 0.05 and ^∗∗^p < 0.01 by Mann-Whitney test, n = 19-24 cells per condition. Error bars represent SEM. Scale bar: (A) 20 μm, (E) 10 μm.

Based on electrostatic potential analyses of the NL crystal structure (Tanaka et al., 2012), we propose that NLs bind HS via positively charged residues that line a canyon formed above the dimerization interface between NL cholinesterase-like domains (Figures 5A and 5B). A single HS chain fits well the dimensions of this canyon in which key charged residues are conserved from *C. elegans* to human (Table S3). Indeed, recombinant mouse NL1 ectodomain bound to a heparin column, and binding was abolished by mutating three Arg or Lys (orange in Figure 5B) to Ala in NL1-RA (Figure 5C). This RA mutation did not affect NL1 dimerization (Figure 5D) nor binding of NL1 to the Nrx LNS domain (Figures S5A–S5C), thus specifically disrupting the heparin/HS interaction. We next tested the significance of HS binding for the synaptogenic activity of all NLs by testing NL1-4 RA mutants in the co-culture assay. The ability of NL1-4 to recruit native Nrx on contacting neurons and to induce presynaptic differentiation was greatly reduced by the RA mutation (Figures 5E–5F and S5D–S5G).

To test the effect of NL1 HS interaction at endogenous synapses, we used sparse knockdown of NL1 in hippocampal cultures, as global loss of function has little effect on excitatory synapse numbers (Kwon et al., 2012). NL1 knockdown resulted in a significant reduction in excitatory synapse density, an effect that was rescued by RNAi-resistant NL1^∗^, but not NL1^∗^-RA (Figures 5G and 5H). To address how NL1-RA influences synapse maturation and function *in vivo*, we used a similar knockdown and replacement strategy by in utero electroporation into layer 2/3 cortical pyramidal cells. When co-expressed with the NL1 knockdown vector, the NL1^∗^-RA mutant resulted in a reduction in spine density relative to NL1^∗^ (Figures 5I–5K). Furthermore, to assess synaptic function while controlling for variability in expression patterns, we performed whole-cell recordings and determined the ratio of mEPSC frequency and amplitude in transfected cells relative to neighbor untransfected cells for each mouse. The NL1^∗^ group had higher mEPSC frequency relative to the NL1^∗^-RA group (Figures 5L–5N; frequency was also higher by individual cell analyses, Figures S5H and S5I). Thus, NL1 requires HS binding for full Nrx interaction and for its function in controlling synapse numbers and function in developing cortex (Figure 5O).

### Nrx HS Modification Controls its Function *in Vivo*

**Figure 6:**
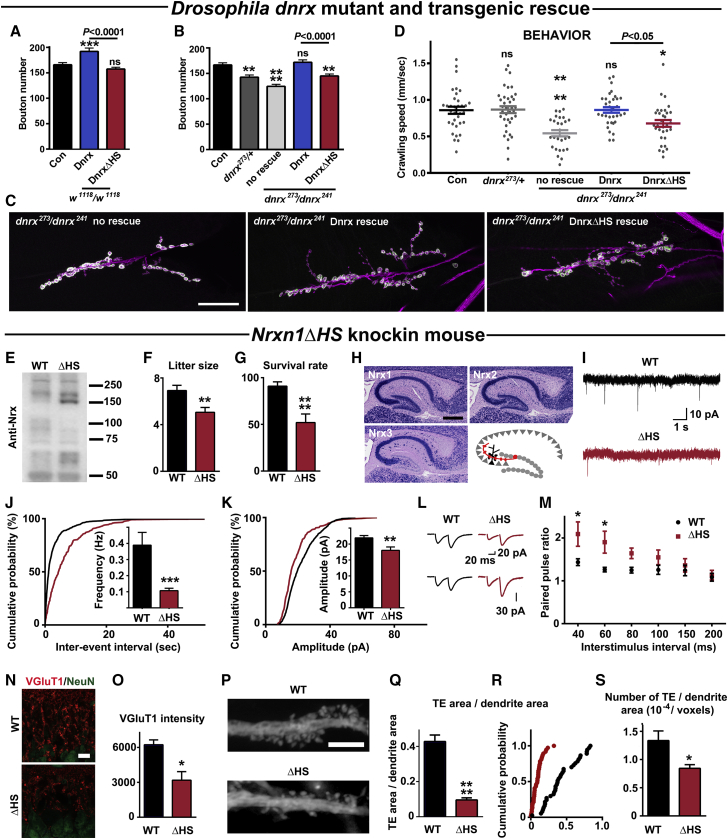
Nrx HS Modification Is Required for its Function *in Vivo* (A) Transgenic overexpression of Dnrx, but not DnrxΔHS induces synaptic overgrowth at *Drosophila* larval muscle 6/7 neuromuscular junctions. (B and C) *Dnrx* mutants showed reduced numbers of synaptic boutons (postsynaptic Discs large in green and neuronal horse radish peroxidase in magenta). Bouton number was rescued by neuronal transgenic expression of Dnrx but not DnrxΔHS. (D) Larval crawling speed was reduced in *dnrx* mutants and rescued by neuronal Dnrx but not DnrxΔHS. (A–D) ^∗^p < 0.05, ^∗∗^p < 0.01, ^∗∗∗^p < 0.001, and ^∗∗∗∗^p < 0.0001 by ANOVA and Bonferroni tests; n = 15–20 (A), 17–31 (B), or 27–37 (D). Full genotypes are as follows. Con = *w*^1118^. Dnrx overexpression = *Elav-Gal4*; *UAS-Dnrx*. DnrxΔHS overexpression = *Elav-Gal4*; *UAS-DnrxΔHS*. *dnrx*^273^/*dnrx*^241^ no rescue = Elav-Gal4; *dnrx*^273^/*dnrx*^241^. *dnrx*^273^/*dnrx*^241^ Dnrx rescue = *Elav-Gal4*; *UAS-Dnrx*; *dnrx*^273^/*dnrx*^241^. *dnrx*^273^/*dnrx*^241^ DnrxΔHS rescue = *Elav-Gal4*; *UAS-DnrxΔHS*; *dnrx*^273^/*dnrx*^241^. (E) *Nrxn1ΔHS* mouse brain homogenate shows a shift in Nrx molecular weight by western blot. (F and G) *Nrxn1ΔHS* mice have reduced neonatal survival indicated by reduced litter size (F). Postnatal survival rates from P0 to P15 are also reduced (G). ^∗∗^p < 0.01 and ^∗∗∗∗^p < 0.0001 by Mann-Whitney test, n = 18–26 litters from homozygous breeding. (H) *In situ* hybridization revealed high expression of Nrx1 and Nrx2 but very low expression of Nrx3 in dentate gyrus granule cells. Mossy fiber synapses (red) from these granule cells to thorny excrescence spines on CA3 pyramidal neurons were studied in panels (I)–(S). (I-K) The frequency and amplitude of mEPSCs in CA3 pyramidal cells was reduced in slices from *Nrxn1ΔHS* mice relative to WT. ^∗∗^p < 0.01 and ^∗∗∗^p < 0.001 by Mann-Whitney test, n = 20 cells from 4 mice each. Inter-event interval and amplitude distributions were significantly different p < 0.0001 by Kolmogorov-Smirnov test. (L and M) Paired pulse ratios of mossy fiber CA3 synapses were elevated in *Nrxn1ΔHS* mice relative to WT. Sample traces are for 40 ms interstimulus interval, with the lower *Nrxn1ΔHS* traces scaled to compare with WT. Genotype p < 0.0001 by two way RM ANOVA with ^∗^p < 0.05 by Bonferroni’s test, n = 11 cells from 4 mice each. (N and O) The integrated intensity of punctate VGlut1 excitatory presynaptic marker was reduced in stratum lucidum, the CA3 region of mossy fiber inputs. NeuN costains CA3 cell bodies. ^∗^p < 0.05 by t test from 3 mice each averaging data from 7 sections per mouse. (P–S) Thorny excrescence (TE) spines were imaged from fluorescent fills of CA3 cells. Total TE area per dendrite shaft area was reduced in *Nrxn1ΔHS* mice relative to WT. ^∗∗∗∗^p < 0.0001 by Mann-Whitney test, n = 37–38 dendrites from 3 mice each; p < 0.0001 by Kolmogorov-Smirnov test of TE area per shaft area distributions. The number of TE spines per dendrite area was also reduced. ^∗^p < 0.05 by Mann-Whitney test, n = 37–38 dendrites from 3 mice each. Error bars represent SEM. Scale bars: (C) 100 μm, (H) 500 μm, (N) 10 μm, (P) 5 μm. See also Figure S6 and Table S3.

**Figure S6:**
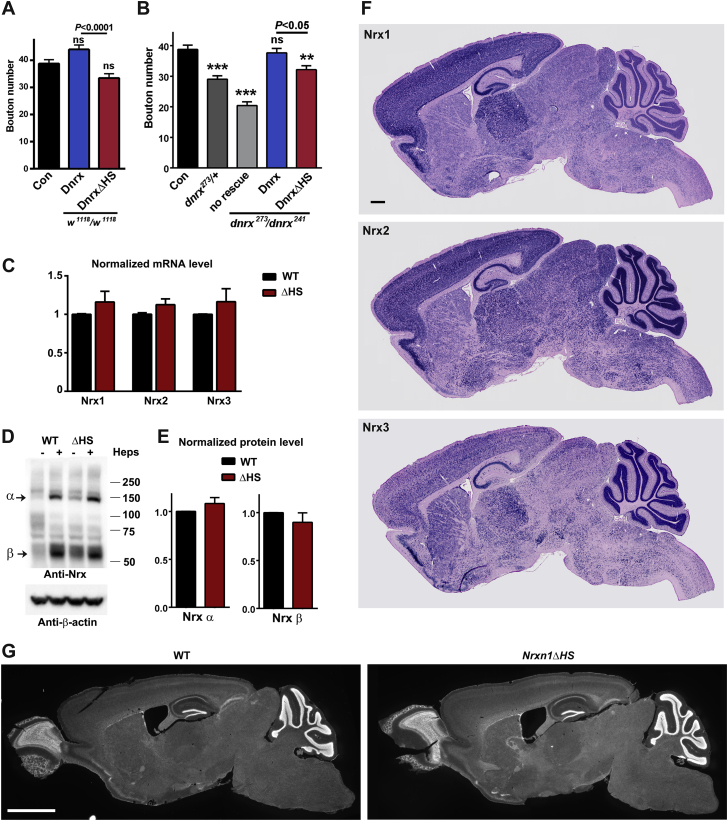
Nrx HS Modification Is Required for its Function *in Vivo*, Related to Figure 6 (A and B) Similar to the findings at larval muscle 6/7 (Figure 4), transgenic overexpression of Dnrx resulted in increased bouton number relative to Dnrx HS at larval muscle 4 neuromuscular junctions (A). *Dnrx* mutants showed reduced numbers of synaptic boutons (B). Bouton number was rescued by neuronal transgenic expression of Dnrx but not DnrxΔHS. ^∗∗^p < 0.01, ^∗∗∗^p < 0.001, and ^∗∗∗∗^p < 0.0001 by ANOVA and Bonferroni tests; n = 17-19 (A), 17-31 (B). Full genotypes are as follows. Con = *w*^1118^. Dnrx overexpression = *Elav-Gal4*; *UAS-Dnrx*. DnrxΔHS overexpression = *Elav-Gal4*; *UAS-DnrxΔHS*. *dnrx*^273^/*dnrx*^241^ no rescue = Elav-Gal4; *dnrx*^273^/*dnrx*^241^. *dnrx*^273^/*dnrx*^241^ Dnrx rescue = *Elav-Gal4*; *UAS-Dnrx*; *dnrx*^273^/*dnrx*^241^. *dnrx*^273^/*dnrx*^241^ DnrxΔHS rescue = *Elav-Gal4*; *UAS-DnrxΔHS*; *dnrx*^273^/*dnrx*^241^. (C) As assessed by quantitative PCR from whole brain, neurexin mRNA levels were not significantly altered in *Nrxn1ΔHS* relative to WT mice (n = 4-5 mice each). (D and E) Mouse brain crude synaptosome fractions were treated with heparinase (Heps) to condense the major Nrx α and β forms (arrows) and processed by western blot (D). Levels of total Nrx α and β were estimated from these major bands relative to the β-actin loading control. Nrx α and β levels were not significantly altered in *Nrxn1ΔHS* relative to WT mice (E, n = 3 mice each). (F) *In situ* hybridization of WT P14 mouse brain showing overlapping expression patterns of neurexins. (G) Gross brain morphology was not obviously different in adult *Nrxn1ΔHS* relative to WT mice as assessed by DAPI nuclear staining. Error bars represent SEM. Scale bar: (F) 500 μm, (G) 2 mm.

Given the conservation of Nrx and its HS modification, we addressed whether Nrx HS modification also regulates Nrx function in *Drosophila*. Null mutants in the single *Drosophila* neurexin *dnrx* survive but exhibit deficits in synaptic structure and function and locomotion behavior (Knight et al., 2011, Li et al., 2007). We confirmed that *dnrx* mutation disrupts locomotion and reduces bouton number at larval neuromuscular junctions, while Dnrx overexpression increases bouton number (Figures 6A–6D and S6A and S6B). We used targeted integrations of transgenes to express equal levels of Dnrx or DnrxΔHS, which lacks HS modification (Figure S1E), under control of a pan-neuronal driver. In contrast to Dnrx, transgenic overexpression of DnrxΔHS in the wild-type (WT) background did not increase bouton number assessed at muscle 6/7 (Figure 6A) or muscle 4 (Figure S6A). Moreover, whereas neuron-specific expression of Dnrx in the *dnrx* mutant background rescued both bouton number and crawling behavior, DnrxΔHS was defective at rescuing both phenotypes (Figures 6B–6D and S6B). Partial rescue observed with DnrxΔHS may relate to the conservation of the HS binding residues among *Drosophila* NL3 and NL4 but not NL1 (Table S3). Thus, Dnrx HS modification is required for normal neuromuscular junction development and locomotion behavior in *Drosophila*.

Finally, to test the role of Nrx HS modification in mice *in vivo*, we generated and analyzed homozygous *Nrxn1ΔHS* mice, which carry point mutations blocking Nrx1 HS modification. Nrx RNA and protein levels did not differ from WT, but there was a shift in the molecular weight distribution of Nrx forms as expected from loss of Nrx1 HS modification (Figures 6E, S6C, and S6D). *Nrxn1ΔHS* mice exhibited reduced litter sizes and reduced survival rates after birth (Figures 6F and 6G). When assessed at adulthood, there was no obvious difference in overall brain morphology of surviving *Nrxn1ΔHS* mice (Figure S6G).

We next studied Nrxn1ΔHS function at the synapses from hippocampal mossy fibers (MFs) of dentate gyrus granule cells to thorny excrescence spines (TEs) of CA3 pyramidal neurons (Figures 6H and S6F). This synapse shows high expression of Nrx1 but little Nrx3, thereby minimizing the possibility of compensation, considering the partially redundant functions of the 3 Nrxs (Missler et al., 2003). mEPSCs recorded from CA3 neurons showed reductions in both frequency and amplitude in slices from *Nrxn1ΔHS* mice relative to WT (Figures 6I–6K). Differences in paired pulse ratio of MF-CA3 synapses also suggest a reduced probability of transmitter release in *Nrxn1ΔHS* mice (Figures 6L and 6M).

To determine whether loss of Nrx1 HS modification affects synaptic structure as well as function, we assessed morphology by immunofluorescence or cellular dye fill and confocal microscopy. The total punctate intensity of presynaptic marker VGluT1 was reduced in the MF synaptic region stratum lucidum of *Nrxn1ΔHS* mice (Figures 6N and 6O). Total TE spine area, as well as the density of TE spines, was reduced along CA3 neuron primary apical dendrites (Figures 6P–6S).

**Figure 7:**
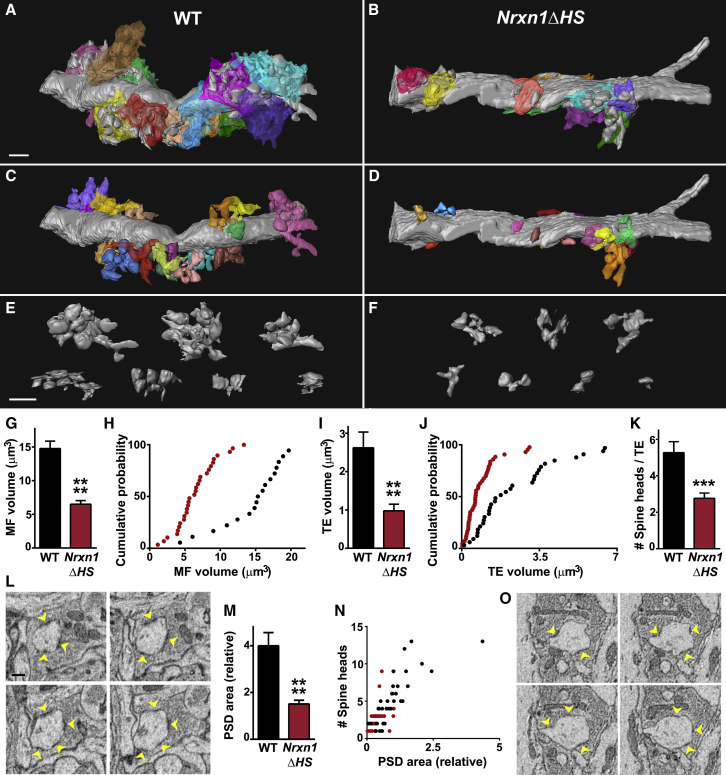
Nrx1 HS Modification Controls Hippocampal Mossy Fiber (MF) and Thorny Excrescence (TE) Synaptic Structure (A-F) SBFSEM reconstructions of CA3 apical dendrite segments and MF inputs revealed differences in synaptic size and complexity in *Nrxn1ΔHS* (B, D, F) relative to WT (A, C, E) mice. (A) and (B) show dendrite segments with TEs in gray and each MF input in a separate color. Panels C and D show each TE on these dendrites in a separate color. (E) and (F) show representative TEs from all reconstructed dendrites. (G and H) MF volume was reduced in *Nrxn1ΔHS* mice relative to WT. ^∗∗∗∗^p < 0.0001 by Mann-Whitney test (G) and p < 0.0001 by Kolmogorov-Smirnoff test (H), n = 18–29 MFs from 2–3 dendrites. (I and J) TE volume was reduced in *Nrxn1ΔHS* mice relative to WT. ^∗∗∗∗^p < 0.0001 by Mann-Whitney test (I) and p < 0.001 by Kolmogorov-Smirnoff test (J), n = 33–43 TEs from 2–3 dendrites. (K) The number of spine heads per TE was reduced in *Nrxn1ΔHS* mice relative to WT. ^∗∗∗^p < 0.001 by Mann-Whitney test, n = 33–43 TEs from 2–3 dendrites. (L–O) PSDs (yellow arrowheads) were visible in WT (L) and *Nrxn1ΔHS* (O) TEs. Estimated PSD surface area per TE was reduced in *Nrxn1ΔHS* mice relative to WT (M). ^∗∗∗∗^p < 0.0001 by Mann-Whitney test, n = 33–43 TEs from 2–3 dendrites. Estimated PSD surface area and the number of spine heads per TE were correlated in both genotypes. Spearman r = 0.877 for WT and 0.626 for *Nrxn1ΔHS*, p < 0.0001 for both genotypes. Error bars represent SEM. Scale bars: (A–D) 2 μm, (E and F) 2 μm, (L and O) 1 μm. See also Figure S7 and Videos S1, S2, S3, and S4.

**Figure S7:**
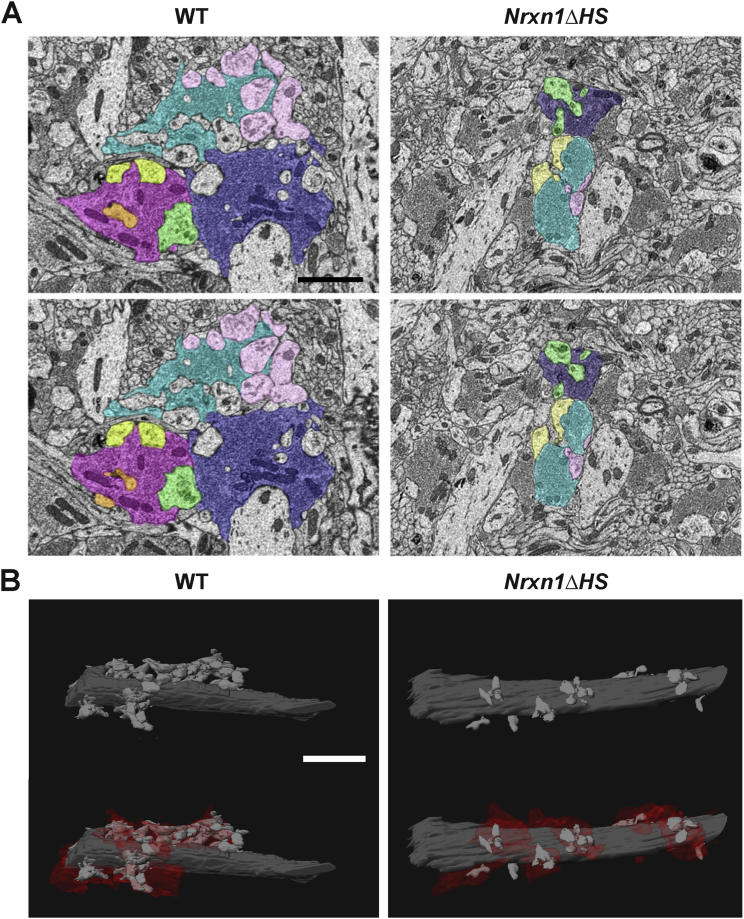
Nrx1 HS Modification Controls Hippocampal Mossy Fiber (MF) and Thorny Excrescence (TE) Synaptic Structure, Related to Figure 7 (A) SBFSEM images showing reconstructed MFs (purple, cyan, and magenta) and TEs (pink, yellow, green, and orange) from hippocampal CA3 stratum lucidum. (B) SBFSEM reconstructions of CA3 apical dendrite segments and MF inputs for different WT and *Nrxn1ΔHS* dendrites than those shown in Figure 6. Dendrite segments with TEs are shown in gray with contacting MFs in red. Scale bars: (A) 1 μm, (B) 5 μm.

We confirmed these pre- and postsynaptic alterations using serial block face scanning electron microscopy (SBFSEM) to visualize MF-CA3 synapses at ultrastructural resolution. Reconstructions of dendritic segments in CA3 stratum lucidum along with their associated MF inputs revealed marked differences in pre- and postsynaptic structures in *Nrxn1ΔHS* mice relative to WT (Figures 7 and S7 and Videos S1, S2, S3, and S4). MF volume and TE volume here for WT mice at 4 weeks postnatal are similar to previous studies of adult mice (Wilke et al., 2013). MF volume and TE volume were both significantly reduced in *Nrxn1ΔHS* relative to WT mice (Figures 7G–7J). The complexity of TEs, assessed by the number of spine heads per TE, was also reduced (Figure 7K). Estimates of PSD surface area per TE correlated with the number of spine heads per TE and were also reduced in *Nrxn1ΔHS* mice relative to WT mice (Figures 7L–7O). Altogether, these studies reveal multiple deficits in function along with significant changes in the size and complexity of pre- and postsynaptic structures of hippocampal CA3 MF-TE synapses in the absence of Nrx1 HS modification.

Video S1. Structure of WT CA3 Apical Dendrite and Associated MF Inputs, Related to Figure 7 A rotating view is shown of the SBFSEM reconstruction of a WT CA3 apical dendrite segment. The TE spines are initially shown with a separate color per spine, and then shown in gray with the MF inputs to these spines shown in separate colors.

Video S2. Structure of *Nrxn1ΔHS* CA3 Apical Dendrite and Associated MF Inputs, Related to Figure 7 A rotating view is shown of the SBFSEM reconstruction of a *Nrxn1ΔHS* CA3 apical dendrite segment. The TE spines are initially shown with a separate color per spine, and then shown in gray with the MF inputs to these spines shown in separate colors. Relative to WT mice (Video S1), the volume and complexity of TE spines and of MFs are reduced in *Nrxn1ΔHS* mice.

Video S3. Structure of WT CA3 Dendritic TE Spines and Associated MF Inputs, Related to Figure 7 A rotating view is shown of the SBFSEM reconstruction of a WT CA3 apical dendrite segment, focusing on three TE spines and their associated MF inputs shown in separate colors.

Video S4. Structure of *Nrxn1ΔHS* CA3 Dendritic TE Spines and Associated MF Inputs, Related to Figure 7 A rotating view is shown of the SBFSEM reconstruction of a *Nrxn1ΔHS* CA3 apical dendrite segment, focusing on three TE spines and their associated MF inputs shown in separate colors. Relative to WT mice (Video S3), the volume and complexity of TE spines and of MFs are reduced in *Nrxn1ΔHS* mice.

## Discussion

### HS Modification of a Core Synaptic Organizer: A Critical Role in Brain Development

We have demonstrated that HS functions centrally as an integral part of the core synaptic organizing complexes Nrx-NL and Nrx-LRRTM. Until now, HSPG functions in the brain were thought to be mediated by members of nine known families of HSPGs, including the membrane-associated syndecans and glypicans and a few secreted HSPGs such as agrin and perlecan, acting mainly as co-receptors and bridging proteins (Farhy Tselnicker et al., 2014, Condomitti and de Wit, 2018). In complement to such roles, our finding that HS is a critical component of Nrxs places HS in a central role in synapse development and function. Furthermore, we show that at least one function previously thought to be mediated by syndecans, presynaptic differentiation triggered by polylysine-coated beads (Lucido et al., 2009), is actually mediated by Nrxs. Our data indicate that the contribution of Nrxs to roles ascribed to other HSPGs needs to be re-evaluated, particularly with respect to synaptic functions.

To the best of our knowledge, the *Nrxn1ΔHS* mouse is the first example of a mouse model in which mutation of a specific HS modification site on one protein impairs mouse survival and synapse development. More generally, glycan modifications of synaptic organizing proteins are typically regulatory and not essential. For example, N glycosylation at splice site B of NL1 regulates interaction with selective forms of Nrx, allowing interaction with β-Nrx, but not α-Nrx, to modulate synapse development (Krueger et al., 2012). Here, we find that HS modification of Nrx is evolutionarily conserved and is essential for normal synapse development in both flies and mice. Electrophysiological recordings, confocal imaging, and SBFSEM at hippocampal mossy fiber CA3 synapses revealed contributions of Nrx1 HS modification to frequency and amplitude of spontaneous transmission, probability of release, synapse numbers, and pre- and postsynaptic structure. These phenotypes, along with the reduced prenatal and postnatal survival of *Nrxn1ΔHS* mice, contrast with the viability and mild phenotypes of mice lacking Nrx1α (Etherton et al., 2009, Missler et al., 2003) or Nrx1β (Anderson et al., 2015), indicating a more critical combined function of Nrx1α+β and, importantly, a critical function of Nrx1 HS modification.

### Integral Glycan and Protein Binding Mode for Canonical Synaptic Organizing Complexes

A surprising finding of this study is that Nrx interactions with its major postsynaptic ligands NL and LRRTM are mediated by HS glycan domains in addition to protein domains. While both the protein and glycan domains of many HSPGs are functionally important, in most cases, this involves binding of one set of ligands to the protein domain and another set of ligands to the glycan domain. Yet here, we find that both domains are involved in the Nrx-NL interaction: the previously identified Nrx LNS protein domain binds the NL acetylcholinesterase-homologous domain, and the newly identified Nrx HS glycan domain binds the NL basic canyon. Based on the effects of mutations in Nrx or NL at either binding site, this dual or cooperative interaction mode involving protein-protein and glycan-protein interfaces between these two transmembrane proteins is required for physiologically relevant function (and, similarly, for Nrx-LRRTM). It is remarkable that the structurally unrelated Nrx ligands NLs and LRRTM1,2 both evolved binding sites for the protein LNS domain and the HS glycan of Nrx. This dual or cooperative interaction mode involving protein-protein and glycan-protein interfaces with a single ligand may not be unique to Nrx, as it may also occur for agrin-laminin and Dally-Decapentaplegic/BMP4 (Cotman et al., 1999, Kirkpatrick et al., 2006). This model is distinct from the role of HSPGs as co-receptors facilitating ligand binding via their glycan domains to separate protein receptors.

It will be important to determine whether Nrx HS modification may positively, or negatively, modulate its interaction with other ligands that bind Nrx protein domains including cerebellins, calsyntenin-3, and C1q-like proteins (reviewed by Südhof, 2017), with further implications for functional interplay among HS-binding or non-HS-binding Nrx ligands. Conversely, our findings that NL1 and LRRTM2 bind HS raise the further possibility that NLs and LRRTM1,2 may bind HSPGs other than Nrx. Such a possibility may be relevant in the context of soluble NL3 ectodomain promotion of glioma growth, where the relevant NL3 receptor is yet to be identified (Venkatesh et al., 2015). Genetic interactions observed among Nrx, NL, an HS-binding growth factor, and an HS modifying enzyme in the control of angiogenesis (Rissone et al., 2012) also point to roles of Nrx as an HSPG and NL as an HS-binding protein outside of their typical synaptic contexts.

### Expanding the Neurexin Interactome

The identification of Nrxs as HSPGs potentially expands the neurexin interactome to hundreds of known HS binding proteins (Table S2). We show that one such HS binding protein, the soluble growth factor pleiotrophin, binds Nrx solely by its HS chain and this interaction can be sufficient to trigger presynaptic differentiation. In mouse models, pleiotrophin regulates synaptic plasticity, spatial learning, and anxiety-like behaviors, at least in part through enhancement of GABAergic transmission (Pavlov et al., 2006), supporting a proposed role for the pleiotrophin-Nrx interaction in regulating synaptic function. Mechanistically, HS-mediated binding of Nrx to such additional ligands may confer key traditional functions of proteoglycans on Nrx, such as to tether soluble molecules, mediate directed diffusion, promote oligomerization, regulate proteolysis, and act as a scaffold and allosteric regulator (Xu and Esko, 2014).

Other synaptic HS-interacting proteins, and thus potential Nrx binding proteins, include type IIA protein tyrosine phosphatases, ephrins, integrins, latrophilin, fibroblast growth factors and their receptors, and glial-derived thrombospondin, which all cooperate with Nrx to promote synaptic differentiation (Christopherson et al., 2005, Hruska et al., 2015, Park and Goda, 2016, Südhof, 2017, Takahashi and Craig, 2013, Terauchi et al., 2010). Indeed, thrombospondin was recently directly isolated as a major Nrx binding protein (Traunmüller et al., 2016). Many such brain-signaling proteins may thus interact with Nrx through their HS modification, unifying synaptic organizing pathways.

### Implications for Brain Function and Dysfunction in Neuropsychiatric Disorders

Given the central role of *NRXN*s and *NLGN*s in a genetically identified synaptic risk pathway in autism, schizophrenia, and a range of neurodevelopmental disorders, our findings have implications for human health. Our data tie mutations in the HSPG pathway into this synaptic pathway. Mutations were found in the essential HS biosynthetic enzymes *EXT1* in autism and *EXTL3* in association with reduced HS concentrations in developmental delay (Li et al., 2002, Oud et al., 2017, Volpi et al., 2017). Mutations not just in the core HS synthetic enzymes but also in HS modifying enzymes are linked to neuropsychiatric disorders. Following initial synthesis of an HS chain, variable deacetylation, epimerization, and N-, 2-O-, 6-O-, and 3-O-sulfation generates diversity, generating thousands of structurally different epitopes (Xu and Esko, 2014). HS structural modifications can be cell-type selective; for example, nine HS sulfotransferases are enriched in distinct GABAergic neuron types (Paul et al., 2017). Genes encoding HS-modifying enzymes include *NDST3* implicated in schizophrenia by a genome-wide association study supported by expression profiling, *NDST1* in which a compound heterozygous mutation was found in developmental delay, and *HS3ST5* associated with autism (Armstrong et al., 2017, Lencz et al., 2013, Wang et al., 2009). Specific HS modification regulates synaptic function in animal models; for example, 6-O-sulfation bidirectionally controls the strength of synaptic transmission at the fly neuromuscular junction (Dani et al., 2012) and controls synaptic plasticity and behavior in mice (Kalus et al., 2009). HS structural motifs generated by HS-modifying enzymes presumably control a range of physiological functions through specific molecular interactions, as demonstrated for some HS motifs (Xu and Esko, 2014). Thus, our findings raise an important direction for future studies, to delineate HS structural motifs on Nrx, uncover cell-type regulation and effects on binding to Nrx ligands, and ultimately, to understand how such HS motifs may contribute to refine synaptic transmission in circuits relevant to neuropsychiatric disorders. Whereas protein-protein interactions are difficult to target therapeutically, the interactions of specific HS structural motifs can be targeted (Xu and Esko, 2014). Thus, our findings raise the possibility of targeting glycan-binding motifs to correct imbalances in the synaptic pathway in neuropsychiatric disorders.

## STAR★Methods

### Key Resources Table

**Table:** 

REAGENT or RESOURCE	SOURCE	IDENTIFIER
**Antibodies**		

Rat anti-HA (3F10)	Roche	Cat#11867431001; RRID: AB_390919
Mouse anti-Myc (9E10)	Santa Cruz	Cat#sc-40; RRID: AB_627268
Rabbit anti-Myc	Sigma	Cat#C3956; RRID: AB_439680
Rabbit anti-GFP	Invitrogen	Cat#11122; RRID: AB_221569
Rabbit anti-pan-Nrx	Millipore	Cat#ABN161; RRID: AB_10917110
Rabbit anti-V5	Millipore	Cat#AB3792; PRID: AB_91591
Mouse anti-HS stub (3G10)	AMSBIO LLC	Cat# 370260; PRID: AB_10892311
Rabbit anti-β Actin	Abcam	Cat#ab8227; PRID: AB_2305186
Mouse anti-Bassoon	Stressgen	VAM-PS003; PRID: AB_10618753
Mouse anti-Synaptophysin	BD Transduction Laboratories	Cat# 611880; RRID: AB_399360
Rabbit anti-Synapsin I	Millipore	Cat#AB1543P; RRID: AB_90757
Mouse anti-vGlut1	NeuroMab	Cat#N28/9; RRID: AB_2187693
Mouse anti-PSD-95	Thermo Scientific	Cat#6G6-1C9; RRID: AB_325399
Guinea pig anti-VGAT	Millipore	Cat#AB5905; RRID: AB_2301751
Mouse anti-Gephyrin	Synaptic Systems	Cat#147021; RRID: AB_1279448
Chicken anti-MAP2	Abcam	Cat#ab5392; RRID: AB_2138153
Mouse anti-Tau	Millipore	Cat#PC1C6; RRID: AB_94855
Rabbit anti-HRP	Cedarlane	Cat#CL7802AP; RRID: AB_2736848
Mouse anti-Dlg	DSHB	Cat#4F3; RRID: AB_528203
Rabbit anti-vGlut1	Synaptic Systems	Cat#135303; RRID: AB_887876
Mouse anti-NeuN	Millipore	Cat#MAB377;RRID: AB_2298772

**Bacterial and Virus Strains**		

AAV6-GFP-4xshRNA	This paper	N/A
AAV6-rNrx-TKD	This paper	N/A

**Chemicals, Peptides, and Recombinant Proteins**		

CNQX	Abcam	Cat#ab120044
DL-APV	Abcam	Cat#ab120271
Bicuculline methiodide	Abcam	Cat#ab120109
Tetrodotoxin (TTX)	Abcam	Cat#ab120054
SR95531 hydrobromide (Gabazine)	Tocris	Cat#1262
(R)-CPP	Tocris	Cat#0247
Tetrodotoxin (TTX)	Tocris	Cat#1069
Nrx1α-AP-Myc-His	This paper	N/A
Nrx1α-ΔHS-AP-Myc-His	This paper	N/A
PTN-hFc	This paper	N/A
hFc	This paper	N/A
HA-NL1-His	This paper	N/A
HA-NL1-RA-His	This paper	N/A
LRRTM2-AP-Myc-His	This paper	N/A
LRRTM2-RA-AP-Myc-His	This paper	N/A
Nrx1β LNS-hFc (Nrx1β-hFc)	This paper	N/A

**Critical Commercial Assays**		

Heparinase I	Sigma	Cat# H2519
Heparinase II	Sigma	Cat# H6512
Heparinase III	Sigma	Cat# H8891
Bacteroides Heparinase I	New England Biolabs	• Cat# P0735L
Bacteroides Heparinase II	New England Biolabs	• Cat# P0736L
Bacteroides Heparinase III	New England Biolabs	• Cat# P0737L
Heparin agarose	GE Healthcare	• Cat# 17-0406-01
BS3-d0 (bis(sulfosuccinimidyl) suberate-d0)	Thermo Fisher Scientific	Cat# 21590
Alexa Fluor 594 Hydrazide	Thermo Fisher Scientific	Cat# A10438

**Experimental Models: Cell Lines**		

Human: HEK293 cells	ATCC	Cat#CRL-1573
Monkey: COS7 cells	ATCC	Cat#CRL-1651
Rat: embryonic day 18 cortical primary neuron culture	This paper	N/A
Rat: embryonic day 18 hippocampal primary neuron culture	This paper	N/A

**Experimental Models: Organisms/Strains**		

Mouse: C57BL/6J	The Jackson Laboratory	JAX: 000664
Mouse: timed-pregnant female C57BL/6	Charles River	Strain code:027
Mouse: *Nrxn1ΔHS*	This paper	N/A
*D. melanogaster w*^1118^	Bloomington Drosophila Stock Center	Stock 3605
*D. melanogaster dnrx*^273^	(Li et al., 2007)	N/A
*D. melanogaster dnrx*^241^	(Li et al., 2007)	N/A
*D. melanogaster Elav-Gal4*	Bloomington Drosophila Stock Center	Stock 458
*D. melanogaster UAS-Dnrx*	This paper	N/A
*D. melanogaster UAS-DnrxΔHS*	This paper	N/A

**Oligonucleotides**		

shRNA targeting sequence: Nrx1 Sh: GTGCCTTCCTCTATGACAACT	(Gokce and Südhof, 2013)	N/A
shRNA targeting sequence: Nrx2 Sh: GAACAAAGACAAAGAGTAT	(Gokce and Südhof, 2013)	N/A
shRNA targeting sequence: Nrx3 Sh: GGCCAGTGAATGAGCATTA	This paper	N/A
shRNA targeting sequence: GFP Sh: GGCGATGCCACCTACGGCAAG	(Alvarez et al., 2006)	N/A
shRNA targeting sequence: NL1 Sh: GGGAAGGGTTGAAGTTTGT	(Kwon et al., 2012)	N/A
shRNA targeting sequence: MorB Sh: GGGAAGGGTTGAAGTTTGT	(Alvarez et al., 2006)	N/A

**Recombinant DNA**		

Nrx1α-CFP	(Siddiqui et al., 2010)	N/A
Nrx2α-CFP	(Siddiqui et al., 2010)	N/A
Nrx3α-CFP	(Siddiqui et al., 2010)	N/A
Amigo-CFP	(Siddiqui et al., 2010)	N/A
Nrx1β-CFP	(Graf et al., 2004)	N/A
Nrx1β-ΔLNS-CFP	(Graf et al., 2004)	N/A
Nrx1β-ΔCHO-CFP	(Graf et al., 2004)	N/A
Nrx1β-ΔCHObeg-CFP	(Graf et al., 2004)	N/A
Nrx1β-ΔCHOend-CFP	(Graf et al., 2004)	N/A
Nrx1β-SA(316)-CFP	This paper	N/A
Nrx1βSSAA(332,333)-CFP	This paper	N/A
Nrx1β-SSSAAA(316,332,333)-CFP	This paper	N/A
pLL3.7-hSyn-V5-Nrx1α	This paper	N/A
pLL3.7-hSyn-V5-Nrx1β	This paper	N/A
pAAV-hSyn-V5-Nrx2α	This paper	N/A
pAAV-hSyn-V5-Nrx2β	This paper	N/A
pAAV-hSyn-V5-Nrx3α	This paper	N/A
pAAV-hSyn-V5-Nrx3β	This paper	N/A
pLL3.7-hSyn-CFP-P2A-V5-Nrx1α	This paper	N/A
pLL3.7-hSyn-YFP-P2A-V5-Nrx1β	This paper	N/A
pLL3.7-hSyn-CFP-P2A-V5-Nrx1α-ΔHS	This paper	N/A
pLL3.7-hSyn-YFP-P2A-V5-Nrx1β-ΔHS	This paper	N/A
pcDNA3.1-Myc-Dnrx	This paper	N/A
pUASTattB-Myc-Dnrx	This paper	N/A
pcDNA3.1-Myc-Dnrx-ΔHS	This paper	N/A
pUASTattB-Myc-Dnrx-ΔHS	This paper	N/A
pNice-HA-NL1	(Scheiffele et al., 2000)	N/A
pNice-HA-NL2	(Scheiffele et al., 2000)	N/A
pNice-HA-NL3	(Graf et al., 2006)	N/A
pNice-HA-NL4	(Graf et al., 2006)	N/A
pNice-HA-NL1-51	This paper	N/A
pNice-HA-NL1-RA	This paper	N/A
pNice-HA-NL2-RA	This paper	N/A
pNice-HA-NL3-RA	This paper	N/A
pNice-HA-NL4-RA	This paper	N/A
HA-LRRTM2	This paper	N/A
HA-CD4	This paper	N/A
NGL-3-CFP	This paper	N/A
pCAGGS-Myc-LRRTM2	This paper	N/A
pCAGGS-Myc-LRRTM2-RA	This paper	N/A
pcDNA4- Nrx1α-PLAP-Myc-His	This paper	N/A
pcDNA4- Nrx1α-ΔHS-PLAP-Myc-His	This paper	N/A
PTN-hFc	This paper	N/A
pcDNA4-HA-ecto-NL1-His	This paper	N/A
pcDNA4-HA-ecto-NL1-RA-His	This paper	N/A
pcDNA4-HA-ecto-NL1-51-His	This paper	N/A
Nrx1β LNS-hFc/ Nrx1β-hFc	(Scheiffele et al., 2000)	Addgene# 59313
LRRTM2-PLAP-Myc-His	(Linhoff et al., 2009)	N/A
LRRTM2-RA-PLAP-Myc-His	This Paper	N/A
pFB-AAV-rNrx-TKD	This paper	N/A
pFB-AAV-GFP-4xshRNA	This paper	N/A
pLL3.7-U6-NL1-shRNA-hSyn-YFP	This paper	N/A
pLL3.7-U6-MORB-shRNA-hSyn-YFP	(Takahashi et al., 2011)	N/A
pFB-hSyn-DIO-YFP-P2A-HA-NL1^∗^	This paper	N/A
pFB-hSyn-DIO-YFP-P2A-HA-NL1^∗^-RA	This paper	N/A
pCMV6-HA-NL1^∗^	This paper	N/A
pCMV6-HA-NL1^∗^-RA	This paper	N/A
pFB-hSyn-DIO-GFP	This paper	N/A
pCAG-Cre	(Matsuda and Cepko, 2007)	Addgene# 13775
pSK(-)-pan-Nrxn1	This paper	N/A
pSK(-)-pan-Nrxn2	This paper	N/A
pSK(-)-pan-Nrxn3	This paper	N/A

**Software and Algorithms**		

Pclamp 10.5	Molecular Devices	https://www.moleculardevices.com/products/axon-patch-clamp-system/acquisition-and-analysis-software/pclamp-software-suite
Fiji 64-bit (ImageJ2)	National Institute of Health	https://imagej.nih.gov/ij/index.html
GraphPad Prism 6	GraphPad Software Inc	https://www.graphpad.com/scientific-software/prism/
MATLab 2014a	MathWorks	https://www.mathworks.com/products/matlab.html
Clustal Omega	Clustal	http://www.clustal.org/omega/
Ilastik version 1.3.0	Ilastik.org	www.Ilastik.org
TrakEM2 1.0a	National Institute of Health	https://imagej.net/TrakEM2
Amira 5.6	Thermo Fisher Scientific	https://www.fei.com/software/amira-3d-for-life-sciences/

### Contact for Reagent and Resource Sharing

Further information and requests for reagents may be directed to, and will be fulfilled by, the Lead Contact, Ann Marie Craig (acraig@mail.ubc.ca). The *Nrxn1ΔHS* knock-in mouse line is covered by an MTA between Taconic Inc. and the University of British Columbia.

### Experimental Model and Subject Details

#### *Drosophila* stocks

Genetic analysis of *dnrx* was performed using the excision alleles *dnrx*^273^ and *dnrx*^241^ (Li et al., 2007). *UAS-dnrx* transgenic flies were generated by targeting pUASTattB-Myc-Dnrx and pUASTattB-Myc-DnrxΔHS vectors to the attp40 landing site during germline transformation. *ElaV-Gal4* was used for Dnrx overexpression and rescue experiments (Lin and Goodman, 1994). Mutant larvae were selected for analysis based on segregation of balancers carrying *actin-GFP*. *w*^1118^ was used as a control strain. For consistency, wandering third-instar female larvae raised at 25°C, 70% humidity were used for all assays. Larvae were procedure- and test-naive for all experiments.

#### Generation of *Nrxn1ΔHS* mice

The *Nrxn1ΔHS* knock-in mouse line was custom generated by Taconic Inc. using homologous recombination. Nrx1 serines 316, 322 and 333 were changed to alanines, corresponding to the SSSAAA mutation described in Figure S1C, by mutation of *Nrxn1* exon 22. The targeting construct was delivered into C57BL/6NTac embryonic stem cells through selection with G418 and Puromycin. Recombination of the targeted allele in ES cells was validated by Southern blot on both 5′ and 3′ ends. Mouse genotyping was performed by PCR analysis and the point mutations were confirmed by sequencing. Initial FRT and LoxP flanked regions were deleted, leaving a single loxP site in the intron 618 base pairs upstream of exon22 in addition to the 3 mutated serines in exon 22.

Primers used for routine genotyping are 13140_35: TGAAGCAACTATAATGTCAGAGAGG and 13140_36: TTTCTACGTGTAGAAATGAAGCAG. The size of the product is 209 bp from wild-type and 267 bp from *Nrxn1ΔHS* knock-in mice. *Nrxn1ΔHS* mice in the C57BL/6NTac background were crossed with C57BL/6J mice (Charles River) for 3 to 6 generations before all the experiments. Due to a low Mendelian ratio from a cross between two heterozygous mice, breeding of homozygous parents was used to generate enough numbers of homozygous mice for analysis. The wild-type mice generated from the same initial *Nrxn1ΔHS* heterozygous crosses were bred in parallel to generate age-matched controls for all experiments. All mice were housed in the Centre for Disease Modeling at the University of British Columbia. Mice were housed in breeding pairs or in same-sex groups of 2-5 with a 12-h light/dark cycle and free access to food and water under specific pathogen-free conditions. Survival rates were not significantly different between males and females and data were pooled. Mice were procedure- and test-naive for all experiments. For consistency, male mice were used for the electrophysiology (P15-17) and electron microscopy (P28-29) assays and female mice for the light microscopy (P21) assays. All procedures were approved by the Animal Care Committee at the University of British Columbia.

#### Electroporated mice

For in utero electroporation, E15.5 timed-pregnant female C57BL/6 mice were directly purchased from Charles River, Massachusetts, United States. Pups were housed with dam until P17-20 when they were used for electrophysiology or imaging experiments. Mice were procedure- and test-naive for all experiments. Data from male and female mice was pooled as gender differences were not found. All procedures for animal surgery and maintenance were performed following protocols approved by the Harvard Standing Committee on Animal Care and in accordance with National Institutes of Health guidelines.

#### Cell culture

Primary rat hippocampal or cortical neuron cultures were prepared from embryonic day 18 rat embryos using previously described methods (Kaech and Banker, 2006). Male and female embryos were pooled for sufficient material. Briefly, 300,000 hippocampal neurons per 60 mm culture dish were grown on coverslips inverted over a glial feeder layer in serum-free media. Cytosine arabinoside (5 μM) was added to hippocampal neuron culture dishes at 2 days *in vitro* (DIV) to prevent overgrowth of glial cells.

COS7 and human embryonic kidney 293 (HEK293) cell lines were maintained in Dulbecco’s Modified Eagle’s medium (DMEM) supplemented with 10% fetal bovine serum (FBS) or bovine growth serum (BGS). COS7 cells (ATCC CRL-1651) are a fibroblast-like cell line derived from male African green monkey kidney tissue. HEK293 cells (ATCC CRL-1573) are thought to be derived from an epithelial or neuronal lineage from female embryonic human kidney tissue. Our cell lines have not been authenticated.

### Method Details

#### Cell culture and co-culture assays

Polylysine bead, pleiotrophin bead and co-culture experiments were initiated at DIV 13. For co-culture experiments, neurons were treated with 100 μM DL-2-amino-5-phosphonovaleric acid (APV) beginning on DIV 7 to limit excitotoxicity.

For knockdown and rescue of Nrx, hippocampal neurons were transfected at DIV 0 using nucleofection (AMAXA Biosystems, Lonza). A mixture of shRNA-resistant CFP-P2A-V5-Nrx1α and V5-Nrx1β at 10:1 ratio was used (Nrx^∗^), or the same amount of shRNA-resistant CFP-2A-V5-Nrx1αΔHS and V5-Nrx1βΔHS at 10:1 ratio (Nrx^∗^ΔHS), or CFP only, all in the same backbone vector pLL3.7-hSyn. For AAV mediated knockdown experiments, neurons on coverslips were exposed to 10^10^ genomic copies of AAV per coverslip for 4 hours at DIV 3 and then returned to their home dishes. The AAV infection was repeated once at DIV 6. mIPSCs were recorded at DIV 13 while mEPSCs were recorded at DIV 14. Neurons were fixed at DIV 14 to investigate endogenous synapse numbers.

For knockdown and rescue of NL1 in neuron culture, plasmids were transfected into primary rat hippocampal neurons at DIV 3 using Lipofectamine 2000. Analysis was performed at DIV 14.

For biochemical experiments in neurons, rat cortical neurons were transfected with V5-Nrx 1, 2, or 3 alpha or β driven by the hSyn promoter using nucleofection. Cortical neurons were plated on six-well plates coated with poly-L-lysine at an approximate density of 900,000 cells/well and cultured in the same medium as hippocampal neurons without addition of cytosine arabinoside. Lysate was harvested at DIV 14.

For co-cultures using COS7 cells or biochemical experiments using HEK293 cells, transfection of those cell lines was performed using TransIT-LT1 Transfection Reagent (Mirus) or 1% Polyethylenimine “Max” from Polysciences, Inc. (24765-1). In co-culture experiments, COS7 cells were trypsinized 24 hours post-transfection and plated onto neurons after two washes with DMEM/FBS, and the co-cultures were maintained for 24 hours before fixation.

#### DNA constructs

All Nrx constructs used for neuron expression and mapping HS modification sites lacked inserts at splice sites 4 and 5, for consistency and to allow co-culture activity with LRRTM2 (Siddiqui et al., 2010). For the biochemical experiments in HEK293 cells in Figure S1B, Nrx1α-CFP, Nrx2α-CFP, Nrx3α-CFP and Amigo-CFP were described previously (Siddiqui et al., 2010). Mouse cDNA of Nrx1β-GFP, Nrx2β-GFP, and Nrx3β-GFP in pEGFP-N1 (Uemura et al., 2010) were generous gifts from Dr. Takeshi Uemura (Shinshu University, Nagano, Japan). These mouse Nrxβ plasmids were used to clone the following regions between HindIII and SpeI in pBluescript SK(-) to generate pSK(-)-pan-Nrxn 1, 2 or 3 plasmids for preparing *in situ* hybridization probes: Nrx1 (GenBank: NM_001346961.1) nucleotides 1430 – 1828, Nrx2 (GenBank: NM_001205235.1) nucleotides 4306 – 4710, and Nrx3 (GenBank: NM_001252074.2) nucleotides 1603 – 1989. These regions were chosen as constitutively expressed regions common to Nrx α and β with minimal homology (22%–43%) between Nrx 1, 2 and 3. For the domain mapping in Figure S1C, Nrx1β-CFP and deletion mutants Nrx1β-ΔLNS-CFP, Nrx1β-ΔCHO-CFP, Nrx1β-ΔCHObeg-CFP, and Nrx1β-ΔCHOend-CFP were described previously (Graf et al., 2004). Nrx1β-SA(316)-CFP, Nrx1βSSAA(332,333)-CFP, and Nrx1β-SSSAAA(316,332,333)-CFP were based on the plasmid above and made by site-directed mutagenesis.

For neuron expression, the cDNAs of Nrx1α-CFP and Nrx3α-CFP were modified by replacing their C-terminal region with the mouse sequence from Nrx1β or Nrx3β. A V5 tag (GKPIPNPLLGLDST) was inserted after the signal peptide of all Nrx constructs and C-terminal CFP or GFP tags were removed. The resulting V5-Nrx1 α and β fragments were inserted into the pLentiLox3.7-hSyn vector to generate pLL3.7-hSyn-V5-Nrx1α and pLL3.7-hSyn-V5-Nrx1β. The DNA fragment of V5-Nrx2 α and β and Nrx3 α and β were cloned into the pAAV-hSyn vector, modified by replacing its CMV promotor with the human Synapsin I promotor, to generate pAAV-hSyn-V5-Nrx2α, pAAV-hSyn-V5-Nrx2β, pAAV-hSyn-V5-Nrx3α and pAAV-hSyn-V5-Nrx3β (used in Figures 1D and 1E). A P2A peptide (GATNFSLLKQAGDVEENPGP) was used to link CFP with V5-Nrx1α or YFP with V5-Nrx1β to make pLL3.7-hSyn-CFP-P2A-V5-Nrx1α and pLL3.7-hSyn-YFP-P2A-V5-Nrx1β. Nrx1 α and β rescue constructs were mutated to be shRNA resistant without changing the protein sequence as indicated by the capital letters: 5′-gtCccAtcGtctatgacTact-3′. Nrx ΔHS mutants were generated by site-directed mutagenesis to bear a single amino acid mutation (LVASAEC to LVAAAEC for Nrx1 α and β, LVASAEC to LVAAAEC for Nrx2 α and β, LVSSAEC to LVSAAEC for Nrx3 α and β). pLL3.7-hSyn-YFP-P2A-V5-Nrx1β and ΔHS mutant were used for Figures. 3G-3J, S2D, S3A and 3B, and S4D-S4I. A 10:1 mix of pLL3.7-hSyn-CFP-P2A-V5-Nrx1α with pLL3.7-hSyn-V5-Nrx1β, and corresponding ΔHS constructs were used for Figures 2 and 3D and 3E, S3C-S3E,S4A and S4B.

*Drosophila* Nrx-1 (Dnrx) cDNA (FlyBase: LP14275) was purchased from the *Drosophila* Genomics Resource Center. A Myc tag (EQKLISEEDL) was inserted after the signal peptide of Dnrx by an overlapping PCR strategy, and then Myc-Dnrx was inserted into pcDNA3.1 or pUASTattB vectors using EcoR1 and Xba1 sites to generate pcDNA3.1-Myc-Dnrx (used in Figure S1E) and pUASTattB-Myc-Dnrx. DnrxΔHS was generated by overlapping PCR to mutate four serine residues to alanine residues (LIFSGAGSGCRGDDEDECTPPFESGSGDD to LIFAGAGAGCRGDDEDECTPPFEAGAGDD) based on the constructs above.

For co-culture experiments, HA-NL1, HA-NL2, HA-NL3, and HA-NL4 in the pNice vector were described (Graf et al., 2004) (these correspond to forms lacking splice inserts for NL2 and NL4, containing the A2 insert for NL3, and containing the A2 and B inserts for NL1). The RA mutations on NLs were generated by site-directed mutagenesis to bear three amino acid mutations (HIGLKPRVKEHYR to HIGLAPAVAEHYR for NL1, HIGLKPRVRDNYR to HIGLAPAVADNYR for NL2, HIGLKPRVRDHYR to HIGLAPAVADHYR for NL3 and HIGLKPRVRDHYR to HIGLAPAVADHYR for NL4). HA-LRRTM2, Myc-LRRTM2, HA-CD4 and NGL-3-CFP, were described previously (Siddiqui et al., 2010, Siddiqui et al., 2013). The Myc-LRRTM2 RA mutant was made by site-directed mutagenesis to change five amino acids from SNSLRTIPVRLFWDCRSLEFLDLSTNRLRSLARNGFA to SNSLATIPVALFWDCRSLEFLDLSTNALASLAANGFA. The resulting Myc-LRRTM2 and Myc-LRRTM2-RA fragments were further inserted into the pCAGGS vector (used in Figures. 5E and 5F, and S5D-5G).

The constructs used for protein purification are listed below. The extracellular part of Nrx1α or Nrx1αΔHS (1-1396) were cloned into the pcDNA4-PLAP-Myc-His vector (Linhoff et al., 2009) for purification of recombinant ectodomain Nrx1α or Nrx1αΔHS used for the carbohydrate analysis and mass spectrometry analysis in Figures 1F and 1G, and S1D. Mouse pleiotrophin cDNA (GenBank: NM_008973) was amplified from mouse brain RNA by RT-PCR and inserted into the pc4-sp-Fc vector (Takahashi et al., 2011) to generate PTN-hFc (used in Figures 1N–1Q, and S2D). The ectodomain of HA-NL1 wild-type or RA mutant (1-697) was fused with a C-terminal his tag (HHHHHH) and then inserted into a pcDNA4 backbone to generate pcDNA4-HA-ecto-NL1 wild-type or RA mutant (used in Figures 3I and 5C and 5D, S4F and S4G). pcDNA4-HA-ecto-NL1-51 mutant was produced by a site mutagenesis strategy to bear three amino acid mutations (IKFMYTDWAD to IKAAYTDAAD). LRRTM2-AP-Myc-His and Nrx1β LNS-hFc (here called the Nrx LNS domain as this Nrx1β-hFc is truncated prior to the region bearing the HS modification site; used in Figures 3J, 6B4C-4E, S4H-S4K, and S5A- S5C) were described previous (Scheiffele et al., 2000, Siddiqui et al., 2010). LRRTM2-RA-AP-Myc-His was made by site mutagenesis based on the wild-type construct (used in Figure 4C).

For the NL1 knockdown and rescue experiment in Figures 5G–5N, a short hairpin RNA (shRNA) sequence 5′-GGGAAGGGTTGAAGTTTGT-3′ from a previous study (Kwon et al., 2012) was ligated into the U6 promoter-driven shRNA expression vector pLL3.7hSyn-YFP to generate pLL3.7-U6-NL1-shRNA-hSyn-YFP. The control shRNA vector pLL3.7-U6-MORB-shRNA-hSyn-YFP was previously described (Takahashi et al., 2011). A P2A peptide (GATNFSLLKQAGDVEENPGP) was used to link YFP with HA-NL1 wild-type or RA mutant first, and these cassettes were inserted with inverted orientations into pFB-hSyn-DIO (Double-floxed Inverted Orientation) backbone (from Virovek Inc.). The resulting constructs were further mutated to be shRNA resistant without changing the protein sequence as indicated by the capital letters: 5′-gCgaGggACtAaagtttgt-3′, which finally generates pFB-hSyn-DIO-YFP-P2A-HA-NL1^∗^ and pFB-hSyn-DIO-YFP-P2A-HA-NL1^∗^-RA. pCAG-Cre (Addgene #13775) was used to activate the expression of NL1 wild-type or RA mutant. pFB-hSyn-DIO-GFP was a gift from Virovek Inc. and used as a control plasmid. The following combination of plasmids were used in the neuron culture experiment: control group (pLL3.7-U6-MORB-shRNA-hSyn-YFP + pFB-hSyn-DIO-GFP + pCAG-Cre at 10:8:2 ratio), knockdown group (pLL3.7-U6-NL1-shRNA-hSyn-YFP + pFB-hSyn-DIO-GFP + pCAG-Cre at 10:8:2 ratio), NL1 rescue group (pLL3.7-U6-NL1-shRNA-hSyn-YFP + pFB-hSyn-DIO-YFP-P2A-HA-NL1^∗^ + pCAG-Cre at 10:8:2 ratio), NL1-RA rescue group (pLL3.7-U6-NL1-shRNA-hSyn-YFP + pFB-hSyn-DIO-YFP-P2A-HA-NL1^∗^-RA + pCAG-Cre at 10:8:2 ratio). The cDNA of HA-NL1^∗^ and HA-NL1^∗^-RA in the rescue constructs was further sub-cloned to replace the cDNA of hNL1 in a pCMV6-hNL1 vector (Kwon et al., 2012) to generate the rescue constructs used in the in utero electroporation experiments. The following combination of plasmids were used in the in utero electroporation experiment: WT rescue group (pLL3.7-U6-NL1-shRNA-hSyn-YFP + pCMV6- HA-NL1^∗^ at 1:1 ratio), NL1-RA mutant rescue group (pLL3.7-U6-NL1-shRNA-hSyn-YFP + pCMV6- HA-NL1^∗^-RA at 1:1 ratio).

#### Generation of Nrx shRNA vector and AAV production

We generated our Nrx triple-knockdown (TKD) AAV vector pFB-AAV-rNrx-TKD (Figure S2B) based on the previously described lentiviral vector L315-Nrx-TKD and control vector L315 (Gokce and Südhof, 2013) which are kind gifts from Dr. Thomas C. Sudhof (Stanford University, Stanford, USA). L315 contains two human H1 and U6 RNA-polymerase III promoters each to express a total of four shRNA sequences. First, we replaced shRNA against mouse Nrx3 from L315-Nrx-TKD with a new sequence targeting all isoforms of rat Nrx3: 5′- GGCCAGTGAATGAGCATTA-3′. Second, the Ubiquitin B promoter and GFP cDNA in L315-Nrx-TKD were replaced with the human Synapsin I promotor. Last, the fragment beginning from the first human H1 promotor to the end of the last poly A from the modified Nrx-TKD vector was inserted between two inverted terminal repeats (ITRs) of the pFB-AAV vector (Chen, 2012) (provided by Virovek). To make control shRNA vector pFB-AAV-GFP-4xshRNA, we replaced four shRNA sequences in pFB-AAV-rNrx-TKD with four identical shRNA sequences against GFP: 5′- GGCGATGCCACCTACGGCAAG-3′ that have no effect on synapse formation and function (Alvarez et al., 2006). pFB-AAV-rNrx-TKD and pFB-AAV-GFP-4xshRNA were packaged by Virovek to generate shNrx (AAV6-rNrx-TKD) and shCon (AAV6-GFP-4xshRNA).

#### Ectodomain fusion protein production

For purification of recombinant Nrx1α wild-type or ΔHS proteins, pcDNA4-Nrx1α-PLAP-Myc-His or pcDNA4-Nrx1αΔHS-PLAP-Myc-His were transfected into HEK293 cells which were cultured in DMEM with 10% FBS and selected for four weeks in the presence of 0.5 mg/ml Zeocin. The resulting Zeocin-resistant cells were grown in a serum-free AIM V synthetic medium for four weeks during which medium was collected every three days.

For the rest of the recombinant proteins in this study, the protein expression constructs were transfected into HEK293 using PEI “Max” (Polysciences, Inc.). After 24 hours post-transfection, the HEK293 cells were cultured in a serum free DMEM medium for 48 hours.

The harvested condition medium was first concentrated in PBS with Centricon Plus-70 filters (Millipore). The recombinant His tag proteins were purified by binding to Ni-NTA agarose beads (QIAGEN) and eluted with 200 mM imidazole. The recombinant Fc tag proteins were purified by binding to protein G beads (GE Healthcare) and eluted with 0.1 M glycine (pH 2.5) followed by a rapid addition of 1/10 volume of TrisHCl (pH 8.6). The purified proteins were washed with PBS and concentrated using Amicon Ultra-15 centrifugal filter units (Millipore) with the appropriate protein cut-off size. Recombinant protein concentrations were quantitated by SDS-PAGE with bovine serum albumin standards using Sypro Ruby gel stain.

#### Glycopeptides and HS compositional analysis

Prior to digestion, the Nrx1α and Nrx1αΔHS ectodomain fusion proteins were reduced with dithiothrietol (Sigma-Aldrich) at a concentration of 5 mM in 50 mM ammonium bicarbonate for 45 minutes at 50°C followed by carboxymethylation with iodoacetamide (Sigma-Aldrich) at a concentration of 15 mM in the dark at room temperature for 45 minutes. This served to open up the tertiary structure for subsequent digestions with Glu-C and *Bacteroides* Heparinases. The samples were dialyzed over 24 hours at 4°C into Milli-Q water (MQ; Barnstead) to remove salts and reagents. The samples were then lyophilized and reconstituted in 50 mM AmBic, and Sequencing Grade Glu-C (Promega) was added in a protease:substrate ratio of 1:100 and digested overnight at 37°C. The next day the digestion was stopped by heating to 100°C for five minutes. 1μL each of *Bacteroides* Heparinases I, II, and III (P0735L, P0736L, P0737L New England Biolabs) were then added to the sample and the mixture was digested at 37°C for 24 hours. After 24 hours another aliquot of *Bacteroides* Heparinases I-III were added to the sample which was further digested for 24 hours. Following *Bacteroides* Heparinase digestions, the resulting heparan sulfate disaccharides were separated from the remaining glycopeptides by passing the sample through a C18 SPE cartridge. The flowthrough contained the heparan sulfate disaccharides, with the peptides and glycopeptides attached to the column. The peptides and glycopeptides were eluted from the column with portions of acetonitrile containing 1% formic acid.

#### HS disaccharide analysis

The resulting heparan sulfate disaccharides collected after *Bacteroides* Heparinase I-III digestion were separated by strong anion exchange (SAX)-HPLC using an analytical HPLC (Agilent 1260 Infinity HPLC, Agilent Technologies) coupled to a post-column heater before being directed back to the HPLC’s FLD fluorescence detector (λex = 346 λem = 410). The eluent from the column was combined with a 1:1 mixture of 0.25 M NaOH (Sigma-Aldrich) and 1% w/v 2-cyanoacetamide (Sigma-Aldrich) pumped at a flow rate of 0.5mL/min through a post-column reactor (SSI Scientific Systems, Inc.) with a 2 mL reactor volume held to a temperature of 130°C before it was directed to the fluorescence detector.

SAX-HPLC was conducted on a 4.6x250mm Waters SAX Spherisorb analytical column with 5 μm particle size at 25°C. Buffer A contained 2.5 mM sodium phosphate, pH 3.5, and Buffer B contained 2.5 mM sodium phosphate, 2 M NaCl, pH 3.5. The gradient began at 3% B and increased to 20% for 10 minutes, before increasing to 25% B over the next 20 minutes, and increasing to 100% B over the next 25 minutes. The gradient stayed at 100% B for 10 minutes, then went back to 3% B for 8 minutes to re-equilibrate the column. The flow rate was 1 mL/min.

Separations of the Heparinase I-III disaccharides were compared to the separations of commercial standard disaccharides (Dextra Laboratories) for identification and calibration.

#### Peptide and glycopeptide analysis by nano-LC-MS

Reduced, carboxymethylated, Glu-C, and Heparinase digested glycopeptides were analyzed with LC-MS. nanoLC-MS/MS was performed on an Orbitrap Fusion Tribrid Mass Spectrometer (ThermoFisher Scientific) coupled to an Ultimate3000 RSLCnano (ThermoFisher Scientific), and equipped with a nanospray ion source. The prepared peptide/glycopeptide sample was resuspended with 100 μL of mobile phase A (0.1% formic acid) and was filtered through 0.2 μm filters (Nanosep, PALL). The sample was injected onto a loading column (volume 10 μL) before being transferred to the separation column (Acclaim PepMap 100 C18 – 0.075x150mm with 2 μm particle size) in 180 minutes on a linear gradient from 100% A (0.1% Formic Acid) to 100% B (80% acetonitrile, 0.1% formic acid) at a flow rate of 0.3 μL/min.

The LTQ was run in automatic mode collecting an MS scan (full FTMS at 150-2000 m/z) followed by data dependent MS/MS scans (CID, HCD, and ETD) at a cycle time of 3 s. The resulting data were searched against the protein sequences using the TurboSequest algorithm (Proteome Discoverer 1.4, Thermo Scientific). The parameters were set to allow 50.0 ppm of precursor ion mass tolerance and 0.8 Da of fragment ion tolerance with monoisotopic mass. Digested peptides were allowed with up to three missed internal cleavage sites, and the differential modifications of 57.02146 Da and 15.9949 Da were allowed for alkylated cysteine and oxidation of methionines, respectively.

The spectra of the glycopeptides in the digest were searched for, and analyzed, manually.

#### Preparation of crude synaptosomal fraction

Brains from mice at different developmental stages were rapidly removed and rinsed once with cold buffer A (320 mM sucrose, 4 mM HEPES-NaOH, pH 7.3). The individual mouse brain was homogenized in 1:10 (weight:volume) buffer A supplemented with protease inhibitor cocktail (Roche) in a glass Teflon homogenizer (nine strokes, 1200 rpm). This homogenate was used directly for the western blots in Figures 6E and S6D, whereas for all other western blots the homogenate was further processed to generate a crude synaptosomal fraction. Homogenate was centrifuged for 10 min at 1000 g twice to remove nuclei. The supernatant S1 was centrifuged for 10 min at 12,000 g. The pellet P2 was resuspended in resuspension buffer (6 mM Tris, pH 8.1, 0.32 M sucrose, 1 mM EDTA, 1 mM EGTA, 1 mM DTT, and protease inhibitors) and centrifuged for 15 min at 14,500 g. The resulting pellet of crude synaptosomal fraction P2ʹ was stored at −80°C.

#### Heparinase treatment

For heparinase treatment of COS7-neuron co-cultures, neurons on coverslips were incubated with or without 0.4 U/ml each of Heparinase I (Sigma, H2519), II (Sigma, H6512), and III (Sigma, H8891) in glia-conditioned medium for two and half hours. COS7 cells were transfected separately with HA or Myc tagged neurexin binding partners or PTP binding partner NGL-3-CFP 24 hours prior to the co-culture. After dissociation in cell dissociation buffer (ThermoFisher Scientific, 13151014), COS7 cells transfected with NGL-3-CFP were mixed with another population of COS7 cells transfected with HA or Myc tagged neurexin-binding partners. The mixed COS7 cells were seeded onto neurons pre-treated with Heparinases I, II, III and the co-culture maintained for 16 hours in the continued presence of heparinases. Under these conditions, COS7 cells expressing NGL-3-CFP served as an internal control on the same coverslip with COS7 cells expressing HA or Myc tagged neurexin partners with heparinase treatment.

For heparinase treatment in biochemical experiments, the proteins in crude synaptosomal fractions or immunoprecipitates bound to protein-G agarose (GE Healthcare Life Sciences) were incubated with or without 1 U/ml each of Heparinases I, II, III for 2 hours at 37°C in the buffer: 20 mM Tris-HCl, pH7, 100 mM NaCl, 1.5 mM CaCl_2_, EDTA-free protease inhibitor cocktail.

#### Heparin binding assay

Heparin agarose (GE Healthcare) was packed into a column and then equilibrated with 20 mM HEPES, pH7.4. Each of the purified recombinant proteins (HA-NL1-His wild-type and RA mutant, and LRRTM2-AP-Myc-His wild-type and RA mutant) was incubated with heparin agarose overnight at 4°C. Bound protein was eluted from the column using a stepwise salt gradient of 50 mM, 100 mM, 150 mM, 200 mM, 250 mM, 300 mM, 350 mM, 400 mM, 450 mM, 500 mM and 1 M NaCl in 20 mM HEPES (pH 7.4). Eluates were collected for each step and probed using immunoblotting.

#### Immunoprecipitation and immunoblotting

HEK or primary neuron cultures were first washed with cold PBS twice and then lysed in cold lysis buffer (50 mM Tris-HCl pH 7.4, 150 mM NaCl, 1% Triton X-100, in some cases with 0.03% deoxycholic acid sodium and 1 mM EDTA) with protease inhibitor cocktail (Roche). To precipitate endogenous Nrx, the P2′ fraction from mouse brains was lysed in the cold lysis buffer directly. Lysate was centrifuged for 30 min at 21,130 g at 4°C. Supernatant was incubated with anti-pan-Nrx (Millipore, ABN161) (Figure 1C), or anti-GFP (Thermo Fisher Scientific, A-11122) (Figures S1B and S1BC) or anti-V5 (Millipore, AB3792) (Figures 1D and 1E), or anti-Myc (Sigma, C3956) (Figure S1EA) antibodies for 2 hours at 4°C. Protein G agarose was added to the lysates and the samples rotated overnight at 4°C. After three washes with lysis buffer, the samples were treated with or without Heparinase I, II, and III for two hours at 37°C. The resulting samples were dissolved in SDS-loading buffer and run on 8% polyacrylamide gels.

Gels were transferred onto Immobilon P membranes which were blocked in 5% skim milk in Tris-buffered saline/ 0.05% Tween-20 for an hour at room temperature followed with one of the following primary antibodies in 3% BSA in Tris-buffered saline/ 0.05% Tween-20: anti-heparan sulfate stub region following heparinase treatment (1:3000; Seikagaku, 3G10), anti-GFP (1:3000; Thermo Fisher Scientific, A-11122), anti-pan-neurexin (1:2000; Millipore, ABN161), anti-V5 (1:5000; Thermo Fisher Scientific, R960-CUS), anti-Myc (1:1000; Santa Cruz, 9E10), anti-β actin (1:10,000; Abcam, ab8227). Membranes were further incubated with secondary antibodies (goat anti-mouse or goat anti-rabbit HRP conjugate from Millipore for chemiluminescence. Immunoblots were detected using the SuperSignal Chemiluminescent kit (Thermo Scientific).

#### Bead induction assays

Polystyrene beads of 7 μm diameter (Bangs Laboratories) were incubated with a solution of poly-D-lysine in sterile PBS (50 μg/ml), overnight at 4°C with end-to-end mixing. Beads were then washed in PBS, resuspended in Neurobasal medium, and added dropwise to the neurons on coverslips (10^5^ beads/coverslip). Uncoated beads were washed in PBS alone and added to the neurons at a density five times that of polylysine-coated beads as they adhered more poorly to the culture coverslips. After 1.5 hours, neuron coverslips were put back to their home glia dishes and cultured for another two days.

To prepare PTN-hFc coated beads, Protein A coated magnetic particles (Spherotech) were incubated with purified PTN-hFc or hFc control for two hours at room temperature. Beads were washed with PBS containing 0.1% BSA using the magnetic separator. The beads were resuspended in conditioned medium and added to the neurons for 1 hour. Neuron coverslips were put back to their home glia dishes and cultured for two days.

#### Protein binding assays

Expression vectors for HA-NL1 wild-type and RA mutant, and Myc-LRRTM2 wild-type and RA mutant were transfected into COS7 cells with PEI “Max.” The next day, the transfected cells were incubated with purified Nrx1β LNS-hFc at the different concentrations in binding buffer (168 mM NaCl, 2.6 mM KCl, 10 mM HEPES, pH7.2, 2 mM CaCl_2_, 2 mM MgCl_2_, 10 mM D-glucose, and 100 ug/ml BSA) for an hour at 4°C. Cells were washed with binding buffer followed by fixation with 4% paraformaldehyde for 12 minutes at room temperature.

For the protein binding on primary neurons, pLL3.7-hSyn-YFP-P2A-V5-Nrx1β wild-type or ΔHS constructs were transfected into the primary rat hippocampal neurons by nucleofection. At DIV 3, neuron coverslips were incubated with conditioned medium containing the purified HA-NL1-His or Myc-LRRTM2-AP-Myc-His proteins for 1 hour in the cell culture incubator on an ice pack to inhibit endocytosis. After binding, the coverslips were washed with conditioned medium followed by fixation with 4% paraformaldehyde.

#### Immunocytochemistry

Neurons and co-cultures were fixed for 12 min with warm 4% paraformaldehyde and 4% sucrose in PBS, pH 7.4. Except for surface staining of non-permeabilized cultures, fixation was followed by permeabilization with 0.2% Triton X-100 for 5 min. Fixed cultures were then blocked in blocking buffer (3% BSA, 5% normal goat serum in PBS) for 30 min at 37°C then incubated in primary antibodies in the blocking buffer overnight at 4°C.

In co-culture experiments, anti-HA (rat, 1:2000; Roche, 11867431001) and anti-Myc (rabbit, 1:8000; Sigma, C3956) were used to stain the surface expression of different ligands on COS7 cells under non-permeabilized conditions. Anti-V5 (1:5000; Thermo Fisher Scientific, R960-CUS) was used to label surface expression of V5-Nrx in the neurons. Anti-pan-Nrx (rabbit, 1:2000; Millipore, ABN161) was used to label recruited neurexins around the beads in polylysine and PTN bead experiments, and in the recruitment assay by LRRTM2 or NL1 expressed in COS7 cells. To assess presynaptic induction, we used anti-bassoon (mouse IgG2a, 1:2000; Stressgen, VAM-PS003), or anti-synaptophysin (mouse IgG1, 1:5000; BD Transduction Laboratories, 611880), or anti-synapsin I (rabbit, 1:5000; Millipore, AB1543P). To analyze endogenous synapse formation in neurons, anti-VGluT1 (mouse IgG1, 1:4000; NeuroMab, N28/9) and anti-PSD-95 (mouse IgG2a, 1:500; Thermo Scientific, clone 6G6-1C9; recognizes PSD-95, PSD-93, SAP102 and SAP97) were used to label excitatory synapses while anti-VGAT (guinea pig, 1:3000; Millipore, AB5905) and anti-gephyrin (mouse IgG1, 1:300; Synaptic Systems, 147021) were used to label inhibitory synapses. Dendrites were identified using anti-MAP2 (chicken polyclonal IgY, 1:8000; Abcam, ab5392) and axons were labeled by anti-tau (mouse IgG2a, 1:2000; Millipore, PC1C6; recognizes dephosphorylated tau).

For all the secondary antibodies, we used highly cross-adsorbed, Alexa-dye conjugated secondary antibodies generated in goat toward the appropriate species and monoclonal isotype (1:1000; Invitrogen; Alexa 488, Alexa-568, and Alexa-647 labeled secondary antibodies). Alexa Fluor 594 conjugated goat anti-human IgG (Fc fragment specific) (1:1000, Jackson ImmunoResearch; 109-585-008) was used to visualize the bound recombinant hFc tagged proteins. AMCA conjugated anti-chicken IgY (donkey IgG; 1:400; Jackson ImmunoResearch; 703-155-155) was used for visualizing dendrites.

Cultures were randomized prior to imaging and quantification so the experimenter was blind to the treatment group. Sets of cells used for quantification were stained simultaneously and imaged with identical settings. Images of cultured cells were acquired on a Zeiss LSM700 or Zeiss Axioplan 2 microscope with 63x/1.4 NA, 40x/1.4 NA and 25x /1.25 NA oil objectives, a Photometrics Sensys cooled CCD or Hamamatsu Orca-Flash4.0 CMOS camera, and custom filters.

For quantification of bassoon, synaptophysin, VGluT1, VGAT or synapsin signals in co-culture or polylysine or pleiotrophin bead assays, cells were selected based on expression of the transfected construct and substantial neurite contact in the phase contrast or DIC channel. Regions were created corresponding to the expressing COS7 cell or bead that excluded MAP2-positive dendrites areas, and the intensity and area of all puncta in the bassoon, synaptophysin, VGluT1, VGAT or synapsin channel were measured. Presynaptic inducing activity was reported as integrated intensity of punctate bassoon per COS7 cell area in Figure 3C, or integrated intensity of punctate synaptophysin per tau-positive axon contact area with beads in Figure 1M, or integrated intensity of punctate VGluT1 or VGAT per bead area in Figure 1Q, or integrated intensity of punctate synapsin per tau-positive axon contact area on COS7 cells in Figures 3E and 4I and 5F.

For quantification of endogenous synapse numbers from primary hippocampal neurons, regions were chosen randomly based on health in the phase contrast channel. Synapses were identified as clusters with pixel overlap between the separately thresholded VGluT1 and PSD-95 channels (excitatory) or VGAT and gephyrin channels (inhibitory). The number of synapses was normalized to the area of MAP2 positive dendrites that were randomly selected from two separate regions per neuron.

#### Neuron culture electrophysiology

mEPSCs and mIPSCs were analyzed by experimenters blind to the treatment group. Recordings were performed at room temperature from neuron cultures at 13-14 days after plating. Neurons were continuously perfused (1 ml/min) with the extracellular solution containing (in mM): NaCl 140, CaCl_2_ 1.3, KCl 5.4, MgCl_2_ 1, HEPES 25, glucose 33 (pH 7.35). mEPSCs were recorded in the presence of 0.5 μM tetrodotoxin and 10 μM bicuculline methiodide; mIPSCs were recorded in the presence of 0.5 μM tetrodotoxin, 10 μM 6-cyano-7-nitroquinoxaline-2,3-dione (CNQX) and 100 μM APV. The patch pipettes were pulled from borosilicate glass capillary tubes (World Precision Instruments) using a PP-830 pipette puller (Narishige). The resistance of pipettes varied between 4-6 MΩ. For mEPSC recording, the patch pipette solution contained (in mM): Cs gluconate 122.5, CsCl 17.5, MgCl_2_ 2, HEPES 10, BAPTA 10, ATP 4, and QX314 5 (pH 7.2). For mIPSC recording, the patch pipette solution contained (in mM): CsCl 140, CaCl_2_ 0.1, MgCl_2_ 2, HEPES 10, BAPTA 10, ATP 4, and QX314 5 (pH 7.2). Neurons were voltage clamped at −60 mV, and recorded with a MultiClamp 700B amplifier (Molecular Devices). Records were filtered at 2 kHz, and acquired with pCLAMP 10 software (Molecular Devices). The series resistance in the recordings varied between 6 to 8 MΩ, and recordings where series resistance varied by more than 10% were rejected. Cells that demonstrated a change in “leak” current of more than 10% (usually less than 50 pA) were rejected from the analysis. No electronic compensation for series resistance was employed. mEPSCs or mIPSCs recorded in a 2 min period were analyzed using Mini 6.0 software (Synaptosoft). The threshold for detection of events was set at approximately 3 times higher than the baseline noise.

#### In utero electroporation and spine imaging

To transfect neocortical layer 2/3 pyramidal neurons, E15.5 timed-pregnant female C57BL/6 mice (Charles River, Massachusetts, United States) were deeply anesthetized with ∼2% isoflurane. Uterine horns were carefully exposed and ∼1 μL of DNA solution (1 μg/μl/plasmid in PBS) were injected into one lateral ventricle. Fast green (0.005%) was added to the DNA solution in order to monitor injection efficiency. Injections were performed with beveled glass micropipettes with ∼50 μm diameter tips (NARISHIGE, Japan). Embryo heads were electroporated with round plate electrodes (0.5 mm diameter) using a BTX ECM830 electroporator (Harvard Apparatus, USA) with 5 pulses (45V, 50 ms) at 1Hz. Warm PBS was dropped onto embryos periodically to prevent drying. The uterus was placed back into the pregnant mouse, and the anterior muscle and the skin were sutured separately. Pups were housed with dam until P17-20 when they were used for electrophysiology or imaging experiments.

Previously electroporated male or female mice were deeply anesthetized with isoflurane and perfused transcardially with 4% paraformaldehyde in 0.1 M sodium phosphate buffer. Brains were fixed for 24 hours at 4°C, washed in phosphate buffer saline (PBS) and sectioned (50 μm) coronally using a vibratome (Leica VT1000S). Brain sections were mounted on glass slides, dried and mounted with ProLong antifade reagent containing DAPI (Molecular Probes). Whole brain sections were imaged with an Olympus VS110 slide-scanning microscope. For dendritic spine analysis high-resolution images of electroporated regions of somatosensory cortex were subsequently acquired with a Keyence BZ-X710 fluorescence microscope with 2D pinhole structure illumination. Confocal stacks of secondary dendrites of L2/3 cortical cells were acquired with a 60x objective and 1 μm spacing in Z. Images were processed and analyzed using ImageJ software.

#### Cortical slice preparation and electrophysiology

Electroporated male or female mice (17-20 days old) were anesthetized by isoflurane inhalation and perfused transcardially with ice-cold artificial cerebrospinal fluid (ACSF) containing (in mM): 125 NaCl, 2.5 KCl, 25 NaHCO3, 2 CaCl2, 1 MgCl2, 1.25 NaH2PO4 and 25 glucose (310 mOsm per kg). Cerebral hemispheres were removed and sliced in cold ACSF (300 μm coronal slices in Leica VT1200S vibratome). Slices were recovered for 15-20 minutes at 34°C in choline-based recovery solution (in mM): 110 choline chloride, 25 NaHCO3, 2.5 KCl, 7 MgCl2, 0.5 CaCl2, 1.25 NaH2PO4, 25 glucose, 11.6 ascorbic acid, and 3.1 pyruvic acid), and then transferred to a holding chamber with 34°C ACSF that progressively cooled down to room temperature (20–22°C). All recordings were obtained within 1-6 h after slicing and solutions were constantly bubbled with 95% O2/5% CO2. Individual slices were transferred to a recording chamber mounted on an upright microscope (Olympus BX51WI) and continuously perfused (1–2 mL per minute) with ACSF at room temperature. Cells were visualized using a 40 × water-immersion objective with infrared DIC optics. Whole-cell voltage-clamp recordings (room temperature) were made from pyramidal cells in L2/3 somatosensory cortex. Patch pipettes (2–4 MΩ) pulled from borosilicate glass (BF150-86-7.5, Sutter Instruments) were filled with a Cs+-based internal solution containing (in mM) 130 CsMeSO4, 10 HEPES, 1.8 MgCl2, 4 Na2ATP, 0.3 NaGTP, and 8 Na2-phosphocreatine,10 CsCl2, 3.3 QX-314 (Cl−salt), (pH 7.3 adjusted with CsOH; 295 mOsm per kg). For all voltage-clamp experiments, errors due to voltage drop across the series resistance (< 20 MOhm) were left uncompensated. To isolate mEPSCs, cells were held at −70mV and ACSF included 20 μM SR95531 hydrobromide (Tocris), 10 μM (R)-CPP (Tocris) and 1 μM Tetrodotoxin (Tocris). Membrane currents were amplified and low-pass filtered at 3 kHz using Multiclamp 700B amplifier (Molecular Devices), digitized at 10 kHz and acquired using National Instruments acquisition boards and a custom version of ScanImage written in MATLAB (Mathworks). Offline analysis of mEPSC frequency was performed using custom routines written in MATLAB and Igor Pro (Wavemetrics). Data from male and female mice was pooled as gender differences were not found. Statistical analyses were done in GraphPad PRISM7 software (GraphPad). mESPC cumulative plots represent the average of 1000 cumulative distributions after random sampling of 80 ISI/Amp values from each recorded cell.

#### Immunohistochemistry and analysis in fly

Wandering third-instar female larvae raised at 25°C, 70% humidity were dissected in PBS and fixed for 20 min in 4.0% formaldehyde. Larval fillets were stained as previously described (Beuchle et al., 2007) using rabbit anti-HRP (1:200, Cedarlane), mouse anti-Dlg (1:50; clone 4F3, DSHB) and fluorescently-labeled secondary antibodies conjugated to Alexa 488 or Alexa 568 (1:200; Invitrogen). Mounted larvae were imaged using a Leica TCS SP5 laser-scanning confocal microscope. Type Ib and Is synaptic boutons at muscle 6-7 and type 1b at muscle 4 were quantified at abdominal segment A2 in intact, fixed larvae.

#### Larval locomotion assay

The larval locomotion assay was adapted from Connolly and Tully (Connolly and Tully, 1998). Individual larvae were placed in the center of a 145-mm diameter Petridish with 2% agar covering the bottom. Larvae were video recorded for 2 minutes and locomotion was analyzed between 30-90 s following introduction onto the dish using custom MATLAB software.

#### *In situ* hybridization

Male or female mice at P14 were deeply anesthetized with pentobarbital and intracardially perfused with 0.1 M phosphate-buffer (PB, pH 7.4) containing 4% (w/v) PFA (4% PFA/PB). Brains were removed from the skull and postfixed with the same fixative overnight at 4°C. After cryoprotection, brains were embedded in OCT compound (Sakura Fine Technical Company, Japan) and cut at 25 μm thickness. Sections were treated with proteinase K (40 μg/ml; Merck). After they were washed and acetylated, sections were incubated with digoxigenin (DIG)-labeled mouse Nrx cRNA probes. The DIG-labeled antisense riboprobes were prepared by transcription of linearized plasmids pSK(-)-pan-Nrxn 1, 2 or 3 using T7 or T3 RNA polymerase and a DIG RNA-labeling kit (Roche). After the sections were washed in buffers with serial differences in stringency, they were incubated with an alkaline phosphatase-conjugated anti-DIG antibody (1:5000; Roche, Japan). The cRNA probes were visualized with freshly prepared colorimetric substrate (NBT/BCIP; Roche, Japan). Nuclear fast red (Vector Labs., Burlingame, CA) was used for counterstaining. Images were acquired on a Zeiss Axio Scan Z1. The Nrx3 image was adjusted for brightness and contrast as the original signal was weaker than for Nrx1 and Nrx2.

#### Hippocampal slice preparation and electrophysiology

For miniature excitatory postsynaptic currents (mEPSCs), coronal hippocampal slices (400 μm) were prepared. Male mice aged P15-P17 were decapitated and the brain was rapidly extracted, after which it was submerged in ice-cold artificial cerebrospinal fluid (ACSF) containing (in mM): 125 NaCl, 2.5 KCl, 2 CaCl_2_, 2 MgCl_2_, 1.25 NaH_2_PO_4_, 26 NaCO_3_, 25 glucose, pH 7.35 (oxygenated with 95% O_2_ and 5% CO_2_, 310-320 mOsm). Slices were cut using a vibratome (VT1000s, Leica). Freshly cut slices were placed in a recovery chamber with carbogenated ACSF at 31°C for at least 30 min and then were kept at room temperature for 60 min before recording. For paired pulse ratios (PPRs), the brain was rapidly extracted and submerged in ice-cold sucrose-based solution containing (in mM): 87 NaCl, 25 NaHCO_3_, 2.5 KCl, 1.25 NaH_2_PO_4_, 7 MgCl_2_, 0.5 CaCl_2_, 25 glucose, and 75 sucrose (oxygenated with 95% O_2_ and 5% CO_2_). Hippocampal slices (400 μm) were cut following Bischofberger et al. (2006) and moved to a heated (31°C) sucrose-based solution containing the same formulation as mentioned above. Slices were allowed to recover for at least 30 min and then were kept at room temperature for 60 min before recording.

Slices were transferred to a recording chamber continuously perfused with carbogenated ACSF (2 mL/min). The patch pipettes were pulled from borosilicate glass capillary tubes (World Precision Instruments) using a pipette puller (P-97, Sutter Instruments). The resistance of pipettes varied between 3-6 MΩ. CA3 pyramidal cells were voltage clamped at −70 mV and the patch pipette solution contained (in mM): Cs gluconate 122.5, CsCl 17.5, MgCl_2_ 2, HEPES 10, BAPTA 10, ATP 4, and QX314 5 (pH 7.2). For mEPSC recordings, tetrodotoxin (1 μM; Abcam) and bicuculline methiodide (10 μM; Abcam) were added prior to recordings to block action potentials and GABA receptor-mediated inhibitory synaptic currents, respectively. For PPR recordings, bicuculline methiodide (10 μM; Abcam) and DL-AP5 (50 μM; Abcam) were used to block GABA receptor-mediated inhibitory synaptic currents and NMDA receptor-mediated excitatory synaptic currents, respectively. Mossy fibers were stimulated electrically in *stratum lucidum* with bipolar tungsten stimulating electrodes (FHC) connected to a constant-current stimulus isolator (DS3; Digitimer). The stimulation intensity was adjusted to obtain both successes and failures. MF origin of recorded input was confirmed by the presence of fast and facilitating EPSCs, as well as by sensitivity to DCG-IV (1 μM; Tocris) at the end of experiments. Recordings were low-pass filtered at 2 kHz, and series resistance was left uncompensated. Cells with changes > 20% in series resistance during recording were discarded.

#### Hippocampal dendritic spine and VGluT1 analysis

To image dendritic spines, we filled CA3 neurons with an Alexa Fluor by microiontophoresis as adapted from a previous study (Dumitriu et al., 2011). P21 female mice were perfused with 4 mL 1% freshly made PFA in phosphate buffer (PB), followed by 45 mL freshly made 4% PFA with 0.125% glutaraldehyde in PB at a rate of 2-3 mL per minute. The brains were dissected out and post-fixed in 4% PFA with 0.125% glutaraldehyde overnight at 4°C, washed in phosphate buffer saline (PBS) and sectioned (200 μm) coronally using a vibratome (Leica VT1000S). All brain sections were then stored in PBS containing 0.02% sodium azide at 4°C. For injection, slices were kept in PBS in a customized circuit module built with an electrode connected to a square pulse stimulator (SD9; ETL). Microinjection pipettes were pulled from borosilicate glass capillary tubes (World Precision Instruments) using a pipette puller (P-97, Sutter Instruments) and backfilled with 2 mM Alexa Fluor 594 Hydrazide (dissolved in 200 mM KCl, Thermo Fisher). Glass pipettes were mounted onto a micromanipulator connected to the same stimulator. The pipette tip was slowly advanced into *stratum pyramidale* of area CA3 in the hippocampus. The movement of the pipette was monitored by a Leica MZ16 F stereomicroscope equipped with a light source and fluorescent filters. The Alexa594 dye was then injected into the cells by step stimuli of 20-60 V.

The dye-filled neurons were imaged on a Zeiss LSM700 confocal microscope with 40x/1.4 NA objective with 2.2x digital zoom. The pinhole size was set to 0.75 AU. The z-step was 300 nm. Image stack were then randomized and blinded to the experimenter for the image quantification. Images were processed with the “Deconvolution” function in the Zeiss Zen Blue software. The classification of spines and dendritic shafts was manually identified first in a few random slices of each stack of images in Ilastik so that all the regions of interest could be identified and segmented by Ilastik. The segmentation was checked manually before it was exported to a binary image stack. The image stack was opened in Fiji from which 3D-volume was measured by “3D Objects Counter.”

Coronal brain sections (200 μm) as prepared above were also used in immunofluorescence staining to quantify vGluT1 intensity in the *striatum lucidum* where granule cell mossy fibers synapse onto CA3 neurons (Figures 6N and 6O). Slices were incubated with primary antibodies anti-vGluT1 (Rb, 1:1000; SySy, 135303) and anti-NeuN (Mouse IgG1, 1:500; Millipore, MAB377) in GDB-Me buffer (30 mM phosphate buffer, pH 7.4, containing 0.2% gelatin, 0.2% BSA, 0.5% Triton X-100, and 0.45 M NaCl) for 48 hours on a shaker at 4°C. After washes with PBS, sections were incubated with secondary antibodies Goat-anti-Rb-Alexa Fluor Plus 488 (1:1000; ThermoFisher Scientific, A32731) and Goat-anti-Mouse IgG1-Alexa Fluor 568 (1:1000; ThermoFisher Scientific, A32731) in GDB-Me buffer overnight at 4°C. Images were collected on a Zeiss LSM700 confocal microscope with a 40x/1.4 NA objective at 2.5x digital zoom at a depth of 6-7 μm into each slice using identical imaging settings. Seven images were collected in striatum lucidum from every animal. Images were then randomized and blinded to the experimenter for quantification. The intensity of vGluT1 was measured in a fixed size rectangular region in Fiji, and the intensity of background staining measured in vGluT1-negative regions from several random images from all animals was subtracted.

To evaluate the overall morphology of mouse brains, adult male mice were intracardially perfused with 4% (w/v) PFA in PBS. Brains were removed from the skull and post-fixed with the same fixative overnight at 4°C. After cryoprotection, brains were embedded in OCT compound (Sakura Fine Technical Company, Japan) and cut at 50 μm thickness. Sections were stained with 1:30,000 DAPI (stock 10 mM, Molecular Probes) for 30 min at room temperature and mounted in Immu-Mount medium (ThermoFisher Scientific). Two DAPI images per brain slice were collected under a Zeiss ApoTome.2 microscope equipped with a Plan-NeoFluar Z 2.3X object and then stitched manually in Photoshop CS5.

#### Serial block face scanning electron microscopy

Male mice aged P28-P29 were anesthetized with 20% urethane and perfused intracardially with 4 mL Ringer’s solution (123 mM NaCl, 1.5 mM CaCl_2_, 5 mM KCl, pH 7.4) with Heparin (20 units/ml) followed by 45 mL 4% paraformaldehyde with 2.5% glutaraldehyde in 0.1 M sodium cacodylate buffer, pH7.4. The brains were immediately removed and placed in the same fixative solution for 3 hours. 500 μm brain slices were cut in 0.1 M sodium cacodylate buffer, using a vibratome (Leica VT1000S). The slices were prepared for Serial block face scanning electron microscopy (SBFSEM) as before (Della Santina et al., 2016). The slices were incubated in a 0.1 M cacodylate buffer (0.66% lead in 0.03M aspartic acid, pH5.5) containing 1.5% potassium ferrocyanide and 2% osmium tetroxide in for 1 hour. After rinsing in buffer, the slices were placed in a freshly made thiocarbohydrazide solution (0.1g TCH in 10 mL double-distilled H_2_0 heated to 60°C for 1 h) for 20 min at room temperature (RT). The slices were rinsed again, incubated in 2% OsO_4_ for 30 min at room temperature (RT), rinsed, and stained *en bloc* in 1% uranyl acetate overnight at 4°C. The slices were then washed, stained with Walton’s lead aspartate for 30 min, washed again, then dehydrated in a graded ice-cold alcohol series, and placed in propylene oxide at RT for 10 min. Finally, the brain slices were embedded in Durcupan resin. The block was then trimmed and mounted on a pin and imaged using an Apreo SBFSEM microscope (Thermo-Fisher).

For wild-type, an area of 33.8 μm X 22.5 μm was imaged at 5.5 nm/x-y-pixel size with a z-depth of 50 nm. For *Nrxn1ΔHS*, an area of 36.9 μm X 24.6 μm was imaged at 6 nm/pixel with a z-depth of 60 nm. Each dataset contained around 1000 serial images. The accelerating voltage was 1.31 kV to 1.783 kV. The TrakEM2 plugin built into Fiji was used to assemble images for 3D reconstruction and analysis (Cardona et al., 2012). Randomly chosen dendrites (2 WT and 3 *Nrxn1ΔHS* dendrites) with their thorny excrescence spines (TEs) and contacting mossy fiber boutons (MFs) were traced using the tracing tools in TrakEM2. TEs were identified as dendritic protrusions with a single spine neck and multiple spine heads, contacted by MFs. MFs were identified as axon enlargements filled densely with synaptic vesicles adjacent to TE spines or the dendritic shaft. Only the vesicle-filled region of MFs was included in the volume estimates. Volumes of TEs and MFs were measured after a 3D reconstruction was generated in TrakEM2. The number of spine heads from each TE was manually counted from the 3D reconstruction in TrakEM2. Postsynaptic densities (PSD) were identified as apparent dense structures in the TE spines apposed to vesicle-rich regions of MF boutons. PSD area was estimated by summing the length of the PSD times the section depth for each component section. All analysis and quantification were done using raw image data. For presentation, raw EM images were smoothed by a Gaussian blur (1.0 pixel) and contrast and brightness adjusted. The surface meshes of 3D reconstructions of all objects were exported to Amira 5.6 (Thermo-Fisher) to adjust the color and transparency of the structures for presentation and for making movies.

### Quantification and Statistical Analysis

Quantification was described in the respective Method Details sections. Statistical details are provided in the figure legends. Analysis was performed by using ImageJ (NIH), Microsoft Excel, and GraphPad Prism 6. In all cases, data were tested for normality using the D’Agostino and Pearson test. If all datasets passed the test, statistical comparisons were made with Student’s unpaired t test or one-way ANOVA with post hoc Bonferroni’s multiple comparison tests, as indicated in the figure legends. Otherwise, nonparametric tests were used. All data are reported as the mean ± standard error of the mean (SEM).

## Supplemental Information

Document S1. Tables S1 and S3

Table S2. HS-Interacting Proteins in the Central Nervous System, Related to Figure 1 Expression in the central nervous system is based on the mRNA expression profile from several databases (Microarray from BioGPS, RNA-seq from Illumina Body Map, and SAGE [Serial Analysis of Gene Expression] available from the GeneCards website).
