# Supplementary material for: Heparan Sulfate Organizes Neuronal Synapses through Neurexin Partnerships
Source: Cell. 2018 Sep 6;174(6):1450–1464.e23. doi: 10.1016/j.cell.2018.07.002 (PMC6173057; doi:10.1016/j.cell.2018.07.002)
Supplement: Document S1. Tables S1 and S3 [file mmc1.pdf]

Cell, Volume *174*

Supplemental Information

Heparan Sulfate Organizes Neuronal Synapses through Neurexin Partnerships

Peng Zhang, Hong Lu, Rui T. Peixoto, Mary K. Pines, Yuan Ge, Shinichiro Oku, Tabrez J. Siddiqui, Yicheng Xie, Wenlan Wu, Stephanie Archer-Hartmann, Keitaro Yoshida, Kenji F. Tanaka, A. Radu Aricescu, Parastoo Azadi, Michael D. Gordon, Bernardo L. Sabatini, Rachel O.L. Wong, and Ann Marie Craig

Table S1. The HS Modification Site on Nrxs is Evolutionary Conserved, Related to Figure 1

(A) Evolutionary conservation in vertebrates.

Nrxn	Species	NCBI/Uniprot	Sequence Alignment
Nrx1	Macaca mulatta	XP_014967815	TTTTLATSTARRGKPPT-KEPISQTTDDILVASAECP—SD-DEDIDPCEPS
Nrx2	Macaca mulatta	XP_014969352	TTTTMATTTTRRGRSPTLRDSTTQNTDDLLVASAECP—SD-DEDLEECEPS
Nrx3	Macaca mulatta	XP_005561989	TTTTMATTTTRKNRSTAS----IQPTSDDLVSSAECS—SD-DEDFVECEP-
Nrx1	Rattus norvegicus	AAA41705	TTTTLATSTARRGKPPT-KEPISQTTDDILVASAECP—SD-DEDIDPCEPS
Nrx2	Rattus norvegicus	AAA41708	TTTTMATTTTRRGRSPTMRDSTTQNTDDLLVASAECP—SD-DEDLEECEPS
Nrx3	Rattus norvegicus	AAA02857	TTTTMATTTTRKNRSTAS----IQPTSDDLVSSAECS—SD-DEDFVECEP-
Nrx1	Bos Taurus	AAA19906	TTTTLATSTARRGNSPT-KEPVSQTTDDILVASAECP—SD-DEDIDPCEPS
Nrx2	Bos Taurus	XP_010819426	TTTTMATTTTRRGRSPTLRDSTTQNTDDLLVASAECP—SD-DEDLEECEPS
Nrx3	Bos Taurus	AAA19908	TTTTMATTTTRKNRSTAS----IQPTSDDLVSSAECS—SD-DEDFVECEPS
Nrx1	Gallus gallus	ACF35428	TTTTLATSTARRGKAPT-KEPIGQTTDDILVASAECP—SD-DEDIDPCEPS
Nrx3	Gallus gallus	ACF35430	TTTTMATTTTRKNRSPPS----IQT-TDDIVSSAECS—SD-DEDFIDCEPS
Nrx1	Xenopus tropicalis	XP_012819327	TTTTLATST---RKSPT-REPVGQTTDDILVASAECP-SDDDEDIDPCEPS
Nrx2	Xenopus tropicalis	XP_004913566	TTTTMATTTTRKGRSPTMRDSITQNSDDLLVASAECP—SD-DEDLEECEPS
Nrx3	Xenopus tropicalis	XP_012824757	TTTTLATTTTRKNRSSPG----STL-TDDIGSSAECFISSDDEDVTDCDT-
Nrx1	Danio rerio	ABG25164	TTSNTI---TIT---YSTPADEQQTTDELLVASAECP—SD-DEDIDPCEPS
Nrx2	Danio rerio	ABG25168	TTTTMATTTTRKQRSPTMRDSVTQNTDDLLVASAECP—SD-DEDLEECEPG
Nrx3	Danio rerio	ABG25174	TTTTLSTTTTRKQRSPPT----IQT-TDDIVSSAECS—SD-DEDLEECDG-
			** **** ** *** *

The sequence surrounding the HS-modified serine (red highlight) is conserved in monkey, rat, bovine, chicken, frog, and zebrafish neurexins, as well as in human and mouse neurexins (Figure 1I). Alignment was performed with Clustal; *, conserved residues.

(B) Potential HS modification in *Caenorhabditis elegans.*

```
VNVNGLMILD LYENGSNRIH TIGAPQTTAV SEQVSSESEE DDELAEMMMA HSIDENPNEA LIESLAPSCL SLEEQQSCFI
DTDDSTGFFS PVLPTVANFP TTRQTEPETT RRPLSPSKMT PSLPSPTTTT LPVFFLSQIT DGDESEDEFD GSGDDLFGGD
GVGITAATQP SVKRTTSISV VTKVLTTTSE PSSTHRSTVL PRPYASVKEA AEQNPDYLGA SIWNQVDTLP EPMVTGPAWR
TNKSLTTTAT TEASSTKSNR KTTTTTTTSA TTTIRIQQRP NYDIDNEVTA LITSSLAPQK TRPKSTPHFT VYPVRPTTPM
GDTITTTMQA ATVTDFPRTP LIMCSSLAVI IAIAAVVFFV
```

The sequence of *Caenorhabditis elegans* neurexin, Nrx-1, between the LNS domain (cyan) and the transmembrane region (green) reveals a potential HS modification site (S, red highlight, flanked by underlined acidic residues).

Table S3. Evolutionary Conservation of the Proposed HS Binding Site on NLs, Related to Figure 6

NL	Species	NCBI/Uniprot	Sequence Alignment
NL1	Human	Q8N2Q7	QLYLHIGLKPRVKEHYRANKVNLWLELVPHLHNLNDISQYT
NL2	Human	Q8NFZ4	KQYLHIGLKPRVRDNYRANKVAFWLELVPHLHNLHTELFTT
NL3	Human	Q9NZ94	QLYLHIGLKPRVRDHYRATKVAFWKHLVPHLYNLHDMFHYT
NL4X	Human	Q8N0W4	QLYLHIGLKPRVRDHYRATKVAFWLELVPHLHNLNEIFQYV
NL4Y	Human	Q8NFZ3	QLYLHIGLKPRVRDHYRATKVAFWLELVPHLHNLNEIFQYV
NL1	Macaca mulatta	XP_011753510	QLYLHIGLKPRVKEHYRANKVNLWLELVPHLHNLNDISQYT
NL2	Macaca mulatta	NP_001265378	KQYLHIGLKPRVRDNYRANKVAFWLELVPHLHNLHTELFTT
NL3	Macaca mulatta	XP_014983140	QLYLHIGLKPRVRDHYRATKVAFWKHLVPHLYNLHDMFHYT
NL4X	Macaca mulatta	XP_014982385	QLYLHIGLKPRVRDHYRATKVAFWLELVPHLHNLNEIFQYV
NL4Y	Macaca mulatta	XP_014984076	QLYLHIGLKPRVRDHYRATKVAFWLELVPHLHNLNEIFQYV
NL1	Rattus norvegicus	Q62765	QLYLHIGLKPRVKEHYRANKVNLWLELVPHLHNLNDISQYT
NL2	Rattus norvegicus	Q62888	KQYLHIGLKPRVRDNYRANKVAFWLELVPHLHNLHTELFTT
NL3	Rattus norvegicus	Q62889	QLYLHIGLKPRVRDHYRATKVAFWKHLVPHLYNLHDMFHYT
NL1	Mus musculus	Q99K10	QLYLHIGLKPRVKEHYRANKVNLWLELVPHLHNLNDISQYT
NL2	Mus musculus	Q69ZK9	KQYLHIGLKPRVRDNYRANKVAFWLELVPHLHNLHTELFTT
NL3	Mus musculus	Q8BYM5	QLYLHIGLKPRVRDHYRATKVAFWKHLVPHLYNLHDMFHYT
NL4	Mus musculus	B0F2B4	QLYLHIGLRPRVRDHYRAAKVAFWLELVPHLHGLAADPGAY
NL1	Bos Taurus	NP_001192902	QLYLHIGLKPRVKEHYRANKVNLWLELVPHLHNLNDISQYT
NL2	Bos Taurus	NP_001178171	KQYLHIGLKPRVRDNYRANKVAFWLELVPHLHNLHTELFTT
NL3	Bos Taurus	AAI23786	QLYLHIGLKPRVRDHYRATKVAFWKHLVPHLYNLHDMFHYT
NL1	Gallus gallus	NP_001192902	QLYLHIGLKPRVKEHYRANKVNLWLELVPHLHNLNDISQYT
NL3	Gallus gallus	ADD52425	QLYLHIGLKPRVRDHYRATKVAFWKHLVPHLYNLHDMFHYT
NL4	Gallus gallus	ADD52421	QLYLHIGLKPRVRDHYRATKVAFWLELVPHLHNLNEIFQYV
NL1	Xenopus tropicalis	ADB12662	QLYLHIGLKPRVKEHYRATKVNLWLELVPRLHVMNDLT---
NL2	Xenopus tropicalis	XP_002944465	KQYLHIGLKPRVKDNYRANKVAFWLELVPHLHELNTGLHTS
NL3	Xenopus tropicalis	ADB12664	QLYLHIGLKPRVRDHYRATKVAFWKHLVPHLYNLHDMFHYT
NL4	Xenopus tropicalis	NP_001165299	QLYLHIGLKPRVRDHYRATKVAFWLELVPHLHNLNEIFQYV
NL1	Danio rerio	NP_001135737	QLYLHIGLKPRVKEHYRANKVNLWLELVPHLHSLNEVSQII
NL2	Danio rerio	XP_005172672	KQYLHIGLKPRIRDNYRANKVAFWLELVPHLHTIHDEIPTY
NL3	Danio rerio	XP_005165239	QLYLHIGLKPRVRDHYRATKVAFWKHLVPHLYNLHDMFHYT
NL4	Danio rerio	XP_021330915	QLYLHIGLKPRVRDHYRATKVAFWLELVPHLHNINELFQYV
NL3	Drosophila	NP_001036685	KKYLNFDTKPKLKNHYRAHRLSFWLNLIPDLHKPGGDNVPA
NL4	Drosophila	NP_001163661	QKYLEIGMKPRIKNHFRAHQLSIWLRLIPELHRAGMEDVIA
NL1	C. elegans	NP_510283	EAYLEITDRPRVKNYYRNAQVGFWNNFIPQLHKNGKETEPV
			: **.: :*:::: :* :: :* .::* *:
NL2	Drosophila	NP_523496	QLYVELGNKANIQSHYRGHKLSMWLNLIPQLHRHFNINDQS
NL1	Drosophila	NP_731172	QAYLNIGIPPTVGYKYRQIYMNFWNKELPDELNQIAAIQEQ

The three Arg or Lys residues mutated here to disrupt HS binding (red highlight) are conserved in human, monkey, rat, mouse, bovine, chicken, frog, zebrafish, and worm NLs, as well as some isoforms of fly NLs. Fly NL2 may have a weaker HS-binding site composed of a Lys and a Gln whereas fly NL1 seems to lack a consensus HS binding site. Alignment was performed with Clustal.
